# Novel Pathways of Oxidative and Nitrosative Inactivation of the Human MGMT Protein in Colon Cancer and Glioblastoma Cells: Increased Efficacy of Alkylating Agents In Vitro and In Vivo

**DOI:** 10.3390/diseases13020032

**Published:** 2025-01-25

**Authors:** Debasish Basak, Agm Mostofa, Hanumantha Rao Madala, Kalkunte S. Srivenugopal

**Affiliations:** 1 Department of Pharmaceutical Sciences, College of Pharmacy, Larkin University, Miami, FL 33169, USA; 2 Office of Bioequivalence/Generic Drug, Center for Drug Evaluation and Research, Food and Drug Administration, Silver Spring, MD 20993, USA; agm.mostofa@fda.hhs.gov; 3 Department of Preclinical Biology, GlycoEra, Newton, MD 02458, USA; rao.madala@glycoera.com; 4 Department of Pharmaceutical Sciences, Jerry H. Hodge School of Pharmacy, Texas Tech University Health Sciences Center, Amarillo, TX 79106, USA; kalkunte.srivenugopal@ttuhsc.edu

**Keywords:** MGMT, DNA repair, homoglutathione disulfide (hGTX), spermine NONOate

## Abstract

**Background:** O^6^-Methylguanine-DNA methyltransferase (MGMT) is a unique antimutagenic DNA repair protein that plays a crucial role in conferring resistance to various alkylating agents in brain tumor therapy. In this study, we exploited the susceptibility of the active site Cys145 of MGMT for thiolation and nitrosylation, both of which inactivate the enzyme. **Methods:** We designed a redox perturbing glutathione mimetic, a platinated homoglutathione disulfide (hGTX) by adding small amounts of cisplatin (1000:10) and used a nitric oxide-donor spermine NONOate. N6022, a potent inhibitor of S-nitrosoglutathione reductase was used to extend the retention of nitrosylated MGMT in tumor cell culture and subcutaneous xenografts. **Results**: Both hGTX and spermine NONOate inhibited MGMT activity in HT29, SF188, T98G, and other brain tumor cells. There was a robust increase in the alkylation-induced DNA interstrand cross-linking, G2/M cell cycle arrest, cytotoxicity, and the levels of apoptotic markers when either of the agents was used with alkylating agents. In the nude mice bearing T98G and HT29-luc2 xenografts, combinations of hGTX and spermine NONOate with alkylating agents produced a marked reduction in MGMT protein and tumor growth delay and regressions. N6022 treatment increased the presence of nitrosylated MGMT for a longer time, thereby extending the DNA-repair deficient state both in cell culture and preclinical settings. **Conclusions:** Our findings highlight the options for redox-driven therapeutic strategies for MGMT and suggest that oxidative and/or nitrosative inactivation of DNA repair in combination with alkylating agents could be exploited.

## 1. Introduction

MGMT or O^6^-Methylguanine-DNA methyltransferase is a widely expressed direct reversal DNA repair protein associated with the mismatch corrections of O^6^-methyl and O^6^-alkylguanine residues [1,2]. GC to AT transition mutations that arise due to the formation of alkylation adducts on the O^6^-position of guanine in DNA by endogenous metabolites (S-adenosyl methionine, nitrosated amino acids, food, and environmental carcinogens, and anticancer treatments) result in a major mutagenic lesion [3]. MGMT protects the genetic fidelity of cells from the genotoxic effects of O^6^-alkylguanine and O^4^-alkylthymine lesions by transferring the alkyl groups to its active-site cysteine145 in an irreversible, stoichiometric, and suicidal reaction, thereby restoring the guanine base in an error-free one-step reaction [2]. Since the alkyl groups are covalently bound to Cys145 in the protein, MGMT is functionally inactivated after each reaction and is degraded through the ubiquitin (ub) proteolytic pathway acting as the first line of defense against alkylation damage to the DNA [4].

MGMT is highly expressed in a majority of human cancers including gliomas, and this elevated DNA repair interferes with the base mispairing and cytotoxic functions of anticancer alkylating agents [5,6]. Tumor resistance manifests because MGMT successfully repairs the O^6^-methylguanine and O^6^-chloroethylguanine lesions induced by methylating agents [temozolomide (TMZ), procarbazine] and chloroethylating agents [1,3-bis-2-chloroethylnitrosourea (BCNU) and CCNU], respectively; the repair action prevents the mutagenic effects and formation of cytotoxic interstrand DNA cross-links in cancer tissues. Therefore, MGMT protein has emerged as a central determinant of tumor resistance to several clinically used anticancer alkylating agents and is an ideal target for biochemical modulation. These observations are particularly relevant for brain tumors because the hydrophobic and blood–brain barrier-crossing alkylating agents remain the drugs of choice for this tumor type. This prompted the development of numerous strategies to reduce tumor MGMT content. Two pseudosubstrate inhibitors, namely O^6^-benzylguanine (BG) and O^6^-(4-bromothienyl)guanine (lomeguatrib) (BTG) potently inhibit the MGMT protein and improve the efficacy of alkylating agents; however, the unacceptable levels of bone marrow toxicity has been a great concern [6,7]. Since hematopoietic stem cells possess very low levels of MGMT, they are more vulnerable to excessive alkylation damage leading to severe myelosuppression [8]. This significant drawback leads to therapy discontinuation, using alkylating agents at sub-therapeutic levels, and necessitates the use of a gene therapy approach involving the transduction of BG-resistant MGMT genes (G156A or P140K) into the hematopoietic stem cells to render the bone marrow resistant to alkylators [9]. However, gene therapy is expensive, requires several visits to the clinic, and has safety and health insurance issues, making it problematic for cancer patients. Therefore, there is an urgent need for novel, efficient, and non-toxic MGMT inhibitors, not only for brain tumors but also for other MGMT-proficient cancers.

The reactive cysteines that exist as thiolate anions in the redox-sensitive proteins play crucial roles in redox signaling. These anionic cysteines that are usually present on the exposed protein surfaces are the preferred targets for oxidation compared to the bulk cysteines with pKa > 8.0. Cys145 which is poised to accept the alkyl groups in the MGMT protein is highly reactive with a pKa of 4.8; this reactive cysteine resides in a basic microenvironment where a lattice of amino acids and a water molecule facilitates deprotonation of the Cys145 to a tholate anion [4,10,11,12]. Earlier, our lab demonstrated that MGMT is subject to various posttranslational modifications namely, phosphorylation [13], sumoylation [14], glutathionylation [15], and ubiquitination [16,17]. These modifications not only affect the stability and activity of the protein but also provide novel opportunities for DNA repair inhibition and efficient anticancer drug discovery.

In our attempts to design rational and catalysis-driven inhibitors for MGMT, we exploited the reactive nature of the active site Cys145 [18] and its susceptibility to thiolation and nitrosylation, both of which inactivate the MGMT. The active site cysteine 145, being highly nucleophilic reacts readily with glutathione (GSH) forming a mixed disulfide linkage [19] and it is also an excellent substrate for nitrosylation [20]. Therefore, we hypothesized that small molecules capable of inducing oxidative stress in cells could inactivate the MGMT protein and sensitize the brain cancer cells to alkylating drugs. As such, we investigated the effects of a stabilized homoglutathione disulfide (hGTX) on human MGMT. Among the nitrosylating agents tested, we found that spermine NONOate is an excellent inhibitor of MGMT, leading to the degradation of the repair protein. Further, we found that by inhibiting the S-nitroso glutathione reductase (GSNOR) [21], MGMT degradation can be enhanced to facilitate a prolonged DNA repair deficiency. S-nitrosoglutathione reductase is a ubiquitous enzyme that reverses S-nitrosoglutathione (GSNO) to GSH restoring the SH groups. To our knowledge, this is the first report to enhance the nitrosylation state of a therapeutic target. These new pathways of MGMT inhibition greatly sensitized gliomas to alkylating agents.

## 2. Materials and Methods

### 2.1. Cell Lines, Chemicals, and Antibodies

Human colon cancer cell line HT29 and glioblastoma cell lines SF188, T98G, GBM6, and GBM10 were purchased from the American Culture Collection (ATCC, Manassas, VA, USA), and Dr. Jann Sarkaria (University of Rochester). The cell lines were grown in growth media recommended by the American Type Culture Collection. HT29 was cultured in McCoy5A (Corning, New York, NY, USA) and glioblastoma cell lines were cultured in Dulbecco’s modified Eagle’s medium (DMEM) (Corning, New York, NY, USA). All growth media were supplemented with 10% fetal bovine serum (Gibco, New York, NY, USA) and antibiotics (Gibco, New York, NY, USA). Monoclonal antibodies to MGMT and actin were purchased from Millipore Corporation (Burlington, MA, USA), whereas anti-cleaved caspase 3 and anti-cleaved poly ADP-ribose polymerase antibodies were purchased from Cell Signaling Technology (Danvers, MA, USA). N6022, a specific inhibitor of S-nitrosoglutathione reductase (GSNOR) was purchased from Cayman Chemical (Ann Arbor, MI, USA). Unless mentioned otherwise, all other chemicals and reagents were purchased from Sigma–Aldrich Company (St. Louis, MO, USA).

### 2.2. Preparation of Platinated Homoglutathione Disulfide (hGTX)

Homoglutathione is a glutathione homolog present along with glutathione in some leguminous plants [22]. In attempts to generate unusual and rational physiologically relevant small molecule oxidants, we sought homoglutathione, which was custom synthesized by the RS Peptide Synthesis Company (Louisville, KY, USA). First, homoglutathione (10–50 mg) was dissolved in sterile water. Then, sodium hydroxide and cisplatin were added to the mixture to achieve a homoglutathione:cisplatin molar ratio of 1000:10. Coordinate bonding of the SH group of GSH with platinum was achieved through oxidation by adding hydrogen peroxide dropwise to a concentration of 0.4 mM. The mixture was gently stirred, and pH was adjusted to 6.0 with a few drops of 1 M HCl. The final product was lyophilized, the chemical structure verified by NMR, dissolved in 1X PBS and stored at −20 °C.

### 2.3. Assay of MGMT Activity

The MGMT activity was measured by transferring the [^3^H]-labeled methyl groups from the O^6^-position of guanine in the DNA substrate to the MGMT protein as described previously [23,24]. The DNA substrate enriched in O^6^-methylguanine was prepared by reacting [^3^H]—methylnitrosourea (GE Healthcare, 60 Ci/mmol) [18]. Briefly, the cell pellets were washed with cold phosphate-buffered saline (PBS), disrupted by sonication in the assay buffer (30 mM Tris–HCl pH 7.5, 0.5 mM dithiothreitol (DTT), 0.5 mM ethylenediaminetetraacetic acid (EDTA), 5% glycerol and 20 μM spermidine) and centrifuged. The extracts (50–150 μg protein) were supplemented with the [^3^H]-DNA (10,000 cpm) and incubated at 37 °C. The reactions were terminated by adding trichloroacetic acid and the DNA substrate was hydrolyzed at 80 °C. Finally, filtration on glass fiber disks was carried out, and the radioactivity present in protein precipitates was quantitated [23,24].

### 2.4. Western Blotting

Immunoblotting was performed using standard procedures involving SDS-PAGE, electrophoretic transfer of proteins to PVDF membranes followed by blocking in 5% dry milk, and incubations with primary antibodies and secondary antibodies linked with horse radish peroxidase [25]. Enhanced chemiluminescence (ECL) was used to detect the protein bands, which were quantitated using densitometry.

### 2.5. Cell Cycle Analysis

About sixty percent confluent cells were treated with 100 μM hGTX for 12 h, followed by the treatment of 100 μM BCNU and 750 μM TMZ. Untreated cells and cells treated with the individual agents alone were used as controls. The cells were allowed to grow for 24 h after the treatments, followed by harvest and fixation in 70% ethanol. The cells were then washed with PBS and resuspended in the presence of RNase (1 μg/mL) and propidium iodide (PI, 50 μg/mL) for 30 min. Cell cycle histograms were generated using a BD FACS Verse flow cytometer.

### 2.6. Assay for Interstrand DNA Cross-Linking in Tumor Cells

T98G cells were pretreated with 200 μM spermine NONOate for 12 h, followed by treatment with BCNU (0–36 h, 100 μM). The cellular DNA was isolated by lysis in TE buffer (10 mM Tris, 1 mM EDTA, pH 8.0) containing 0.5% SDS, and 100 μg/mL RNase A and proteinase K (1 mg/mL) digestion. Ethanol was used to precipitate the DNA which was then dissolved in TE buffer. The drug-induced DNA cross-linking was measured by the ethidium bromide fluorescence assay as described previously [26]. Briefly, 5–10 μg DNA was dissolved in assay buffer (20 mM potassium phosphate and 2 mM EDTA, pH 11.8) in duplicate. One set of tubes was heated at 100 °C for 10 min and cooled to room temperature. Ethidium bromide was added to 1 μg/mL, and the fluorescence was measured (305 nm excitation and 585 nm emission) using an LS-50 variable wavelength spectrofluorometer (Perkin Elmer). The fluorescence enhancement observed in drug-treated cells was used to compute the cross-link index [26].

### 2.7. Detection of ROS Generation

Intracellular ROS production was determined by 2′,7′–dichlorofluorescin diacetate (DCFDA) staining followed by fluorescence detection using a Biotek plate reader (Model-Synergy 2SL) as described previously [27]. The excitation and emission wavelengths were set at 485 nm and 535 nm, respectively. Briefly, cells were incubated with 10 μM DCF-DA solution at 37 °C for 0.5 h, washed with PBS twice, treated with different concentrations of hGTX, and spermine NONOate for 3 h followed by fluorescence measurement.

### 2.8. Cell Survival Assays

For cell viability assays, the yellow tetrazolium dye [(3-(4,5-dimethylthiazolyl-2)-2, 5-diphenyl tetrazolium bromide) (MTT)] was used. The cells were seeded at a density of 7000 cells per well in 96-well plates and they were allowed to grow for 24 h. They underwent treatment with hGTX alone at concentrations specified for 24 h, followed by exposure to BCNU or temozolomide. In other experiments, cells were treated with spermine NONOate and/or N6022 followed by TMZ. The wells were washed and MTT (160 μL of 2 mg/mL) was added. The plates were incubated for 4 h following which 800 μL of dimethyl sulfoxide was added. The plates were stored at room temperature in the dark for 2 h before reading at 570 nm using a SPECTRAFluor Plus plate reader (Tecan).

### 2.9. Tumor Xenografts and Drug Efficacy Studies

Female nu/nu immunocompromised mice (25 g) were obtained from Charles River Laboratories (Wilmington, MA, USA) and fed ad libitum. They were allowed to acclimatize to a 12 h light–12 h dark cycle and all procedures were performed under the guidelines of the Institutional Animal Care and Use Committee (IACUC). Subcutaneous human tumor xenografts were established according to our previous studies [28]. In the first study, T98G (6.0 × 10^6^) cells in 0.1 mL of media and matrigel (1:1) were injected subcutaneously into the right flanks. Tumor volume was calculated every 2–3 days with a caliper using the following formula: volume = [length × (width)^2^]/2. When the tumors reached a volume of 75–100 mm^3^, the animals were administered 100 mg/kg/day of hGTX, 1 mg/kg/day of cisplatin, 0.2 mg/kg/day of BCNU, and combinations of hGTX either with BCNU or cisplatin at the same dosage regimens. Control mice with tumors received the vehicle alone (PEG-300:ethanol: saline, 60:30:10 v/v) alone. In the second study, HT29-luc2 (5.0 × 10^6^) cells in 0.1 mL of media and matrigel (1:1) were injected subcutaneously into the right flanks, and the same formula for calculating tumor volume was followed. The tumor growth in the luciferase-expressing subcutaneous HT-29 xenografts was measured by non-invasive in vivo bioluminescence measurements using an IVIS-200 Caliper Imaging System. The animals lying in the prone position were given i.p. injections of D-luciferin (2 mg in 100 μL PBS per mouse) and then imaged as described previously [27]. The tumor-bearing mice were administered 100 mg/kg/day of hGTX, 150 mg/kg of NCX4016 4 times weekly, 0.2 mg/kg/day of BCNU, 50 mg/kg of TMZ 4 times weekly, and combinations of hGTX and NCX4016 (m-nitro-aspirin) with the alkylating agents maintaining the same dosage regimens. In the third study, T98G (6.0 × 10^6^) cells in 0.1 mL of media and matrigel (1:1) were injected subcutaneously into the right flanks for xenograft development. The mice were administered 0.4 mg of spermine NONOate intra-tumorally twice a week, 50 mg/kg of TMZ i.p 3 times weekly, 0.1 mg/kg/day of N6022 i.p twice weekly, and combinations of these agents maintaining the same dosage regimens. The animals were sacrificed after 3 to 4 weeks of drug administration. In all three studies, tumors were excised, washed, and homogenized using a polytron in 50 mM Tris–HCl buffer (pH 8.0) containing 3% glycerol, 1 mM ethylenediaminetetraacetic acid, 0.5 mM phenylmethylsulfonyl fluoride, 2 mM benzamidine, 0.5% Triton X—100, and 1 mM sodium vanadate. The samples were centrifuged and the resulting extracts were used for Western blot analyses and MGMT assays.

### 2.10. Hematoxylin and Eosin Staining of Tissue Sections

The major organs collected from the mice with HT29-luc2 xenografts were fixed in 10% formalin, embedded in paraffin, and cut into 5 μm thick sections. The sections were soaked in xylene and ethanol and stained with hematoxylin–eosin (H&E) for morphological evaluation. Images were acquired using an IX81 fluorescence microscope (40X objective) equipped with a CCD camera. The original, unmodified images were used for semi-quantitative evaluation.

### 2.11. Statistical Analysis

All experiments including the Western blot and drug efficacy studies in human xenografts were performed three times independently. The results in repeat experiments were consistent but with minor variations. The differences among control and treatment groups were tested using unpaired, two-tailed *t*-tests for comparison of two means or ANOVA for comparison of three or more groups. When necessary, the sample numbers were adjusted to maintain a power of 0.8 to detect a 40% difference between the groups. For animal numbers, the power analysis was used to achieve a statistical power of >80%.

## 3. Results

### 3.1. Overview of Compounds Used for Redox-Driven Inactivation of Human MGMT

**Figure 1:**
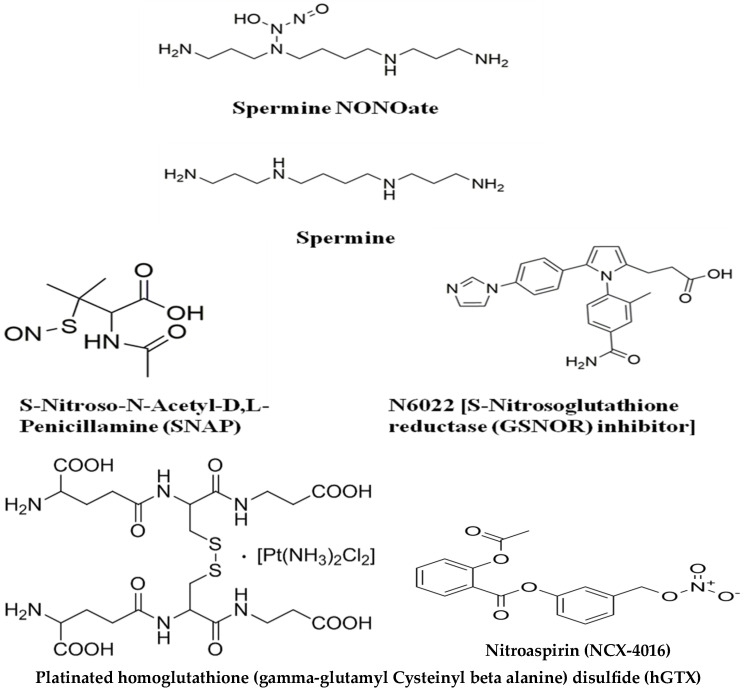
Structures of Cysteine reactive nitrosylating and thiolating agents used in this study to inactivate human MGMT.

In designing redox-altering small molecules that resemble endogenous compounds, we were inspired by NOV-002 or glutoxim, a mimetic of oxidized and stabilized glutathione made of glutathione disulfide in a 1000:1 ratio with cisplatin [29]. NOV-002 is not a substrate for glutathione reductase and has been shown to cause redox imbalance through inhibition of glutathione metabolic enzymes, induction of protein thiolation, and alterations of redox signaling [30,31]. Glutoxim has gone through extensive clinical trials in Russia and the USA as a chemotherapy modulator for cancer and other diseases [32]. Along the lines of NOV-002, we designed hGTX by forming a stabilized homoglutathione disulfide. Homoglutathione with an extra methylene group in its structure is a homolog of glutathione and the two tripeptides coexist in some higher plants [22]. The structures of hGTX and other compounds used for redox-driven inactivation of MGMT are shown in Figure 1. hGTX showed properties such as glutathionylation of bulk proteins and induction of oxidative stress similar to NOV-002 (Unpublished). Nitric oxide donor drug development has drawn much attention because of its therapeutic potential, particularly in cardiac diseases [33]. Several NONOates have been engineered and tested in experimental settings. Here, we chose a NONOate conjugated with an endogenous polyamine, spermidine. Nitroaspirin (NCX-4016; 3-nitro-2-hydroxybenzoic acid acetylsalicylic ester), also called a fatty aspirin, is a well-characterized chemopreventive agent without the GI adverse effects of aspirin and is degraded by plasma and tissue esterases to release NO in a sustained manner [34]. In earlier studies, we characterized the nitrosylation of MGMT and consequent degradation by NCX-4016 and its ability to potentiate the efficacy of clinically used alkylating agents [35,36,37].

N-6022 (1-[4-(aminocarbonyl)-2-methylphenyl]-5-[4-(1H-imidazol-1-yl)phenyl]-1H-pyrrole-2-propanoic acid) is a tight binding potent inhibitor of S-Nitrosoglutathione Reductase (GSNOR) with an IC_50_ of 8 nM [21]. This enzyme reduces S-nitrosoglutathione (GSNO) to oxidized glutathione (GSSG) and ammonia using NADH [38]. This reaction is a key part of the denitrosylating enzymatic system that controls cellular S-nitrosation levels.

### 3.2. Treatment with hGTX and Other Nitrosylating Agents Both Alone and in Combination with Alkylating Drugs Resulted in MGMT Inhibition and Loss of MGMT Protein in Glioblastoma Cell Lines

**Figure 2:**
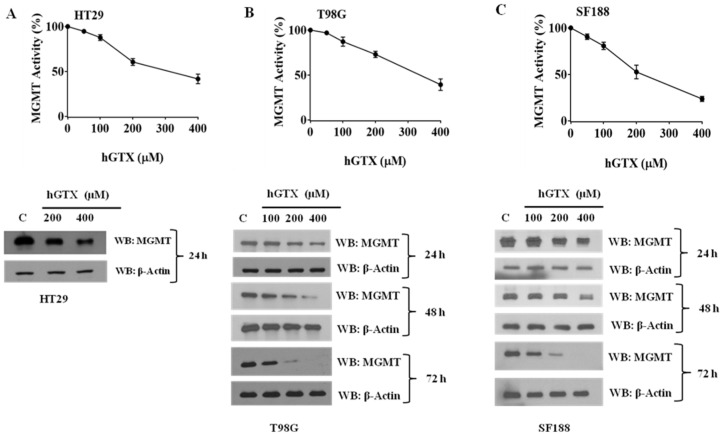
Concentration-dependent inhibition of MGMT activity and MGMT degradation induced by hGTX in human colon cancer and brain tumor cells. Modification of the active site Cys145 in MGMT inactivates the DNA repair and the resulting protein undergoes degradation. (**A**) Inhibition of the DNA repair activity of cellular MGMT by increasing hGTX concentrations. HT29 cells were first treated with hGTX at concentrations shown for 24 h. Cell extracts prepared therefrom were used for determining the DNA repair activity of MGMT (upper panels) and the loss of MGMT protein by Western blot analyses. (**B**) T98G cells; (**C**) SF188 cells. (Whole blot See Figure S1).

**Figure 3:**
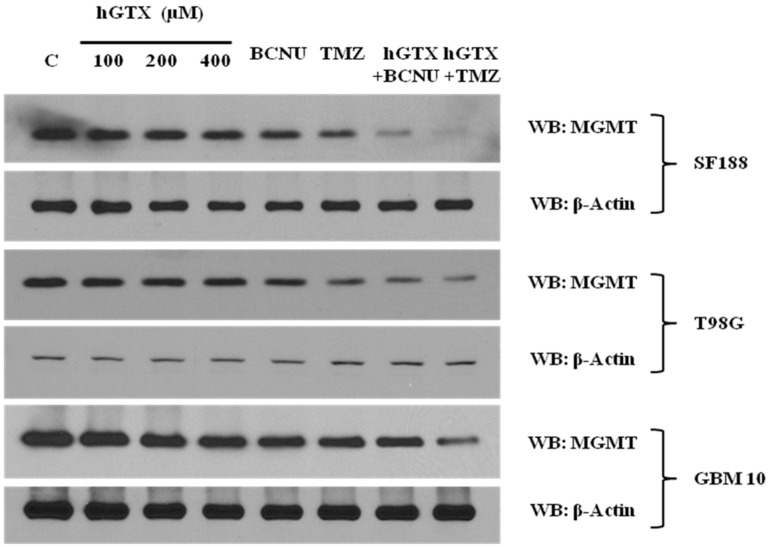
MGMT deficiency induced by hGTX alone and hGTX + alkylator combinations in glioblastoma cell lines. Tumor cells were treated with either hGTX alone or preincubated with 100 μΜ hGTX followed by BCNU (100 μM) and TMZ (750 μM) for 24 h. The results show a marked downregulation of MGMT protein. (Whole blot See Figure S2).

**Figure 4:**
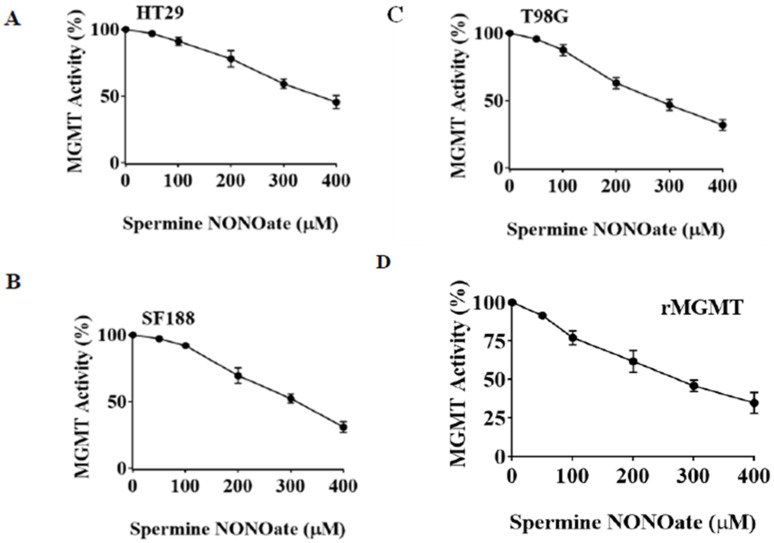

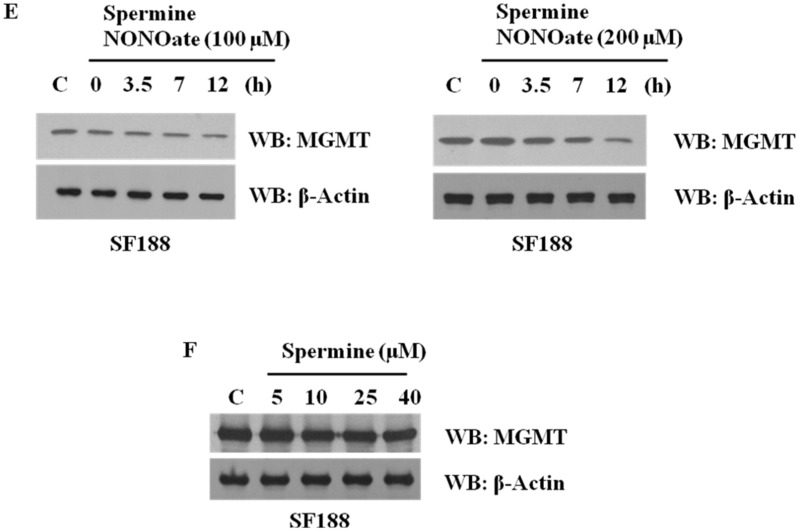
Concentration-dependent inhibition of MGMT activity and MGMT degradation induced by spermine NONOate in human colon tumor and brain tumor cells. (**A**) HT29 cells; (**B**) SF188 cells; (**C**) T98G cells. Tumor cell monolayers were exposed to spermine NONOate at increasing concentrations (0–400 μM) for 24 h. Extracts prepared from these cells were assayed for the DNA repair activity of MGMT. Inhibition of MGMT in all cell lines is evident. (**D**) purified histidine-tagged recombinant MGMT protein (rMGMT, 1 μg) was exposed to spermine NONOate for 10 min and the DNA repair activity was measured. Inhibition of MGMT observed here reflects a direct effect of the nitrosylating agent on the protein. (**E**) Decreased levels of MGMT protein following SF188 cell treatment with spermine NONOate. The extracts used for MGMT activity (**A**–**D**) were Western blotted to determine the protein levels. (**F**) SF188 cells were exposed to spermine as a control and the extracts were immunoblotted for MGMT. The lack of changes indicates that the nitrosylation by the NONOate group was responsible for the degradation of MGMT protein. (Whole blot See Figure S3).

Our previous findings that hGTX can modulate GSH metabolism, increase protein glutathionylation, and augment oxidative stress in human tumors (unpublished) led to the current study investigating the inhibitory effects of stabilized glutathione mimetic on MGMT in brain tumor cell lines. Initial experiments involved the treatment of MGMT-proficient HT29, SF188, T98G, and GBM10 cells with hGTX and quantitation of MGMT activity. In all cell lines, the guanine dealkylation activity of MGMT was inhibited by hGTX in a dose- and time-dependent manner with around 80% inhibition of MGMT activity at 400 μM hGTX (Figure 2A–C). MGMT activity assays showed a gradual loss of activity, consistent with the extent of inhibition after 24 h. Concurrent with the inactivation, MGMT protein was eliminated from the tumor cells (Figure 2 Western blot). MGMT protein was further degraded when GBM cells were treated with hGTX and different alkylating agents. Combinations of hGTX with BCNU and TMZ were more potent in this property with >90% elimination of the MGMT protein in T98G and SF188 cells after 24 h treatment (Figure 3). We also exposed GBM10 cells to hGTX and its combinations with alkylating agents similarly; this cell line, however, showed a modest loss of MGMT protein. Next, the cell lines were treated with spermine NONOate as a single agent for 24 h and the MGMT activity was measured. The nitrosylating agent was able to curtail the activity significantly with an approximate 70% inhibition in most cell lines. A sustained and potent inhibition of DNA repair activity by spermine NONOate was confirmed using the recombinant MGMT protein as well (Figure 4A–D). Loss of MGMT protein from tumor cells also occurred after spermine NONOate treatment, but not with spermine showing the specificity (Figure 4E,F). When tumor cells were preincubated with N6022 (a GSNOR inhibitor) and then exposed to NONOate, the inhibition was even greater, around 80%. These results confirmed that hGTX and other nitrosylating agents are capable of inactivating the MGMT protein and leading to its breakdown in human glioblastoma cells. Taken together, the data suggests that the thiolating and nitrosylating actions of hGTX and NONoate, respectively, cause a rapid inactivation of MGMT and create a DNA-repair deficient state in cells, which can promote the alkylation DNA damage by therapeutic drugs.

### 3.3. MGMT Inhibition by Spermine NONOate Markedly Increases the Alkylation-DNA Damage and Greatly Potentiated the Cell-Killing in a Nitrosylation-Dependent Manner

**Figure 5:**
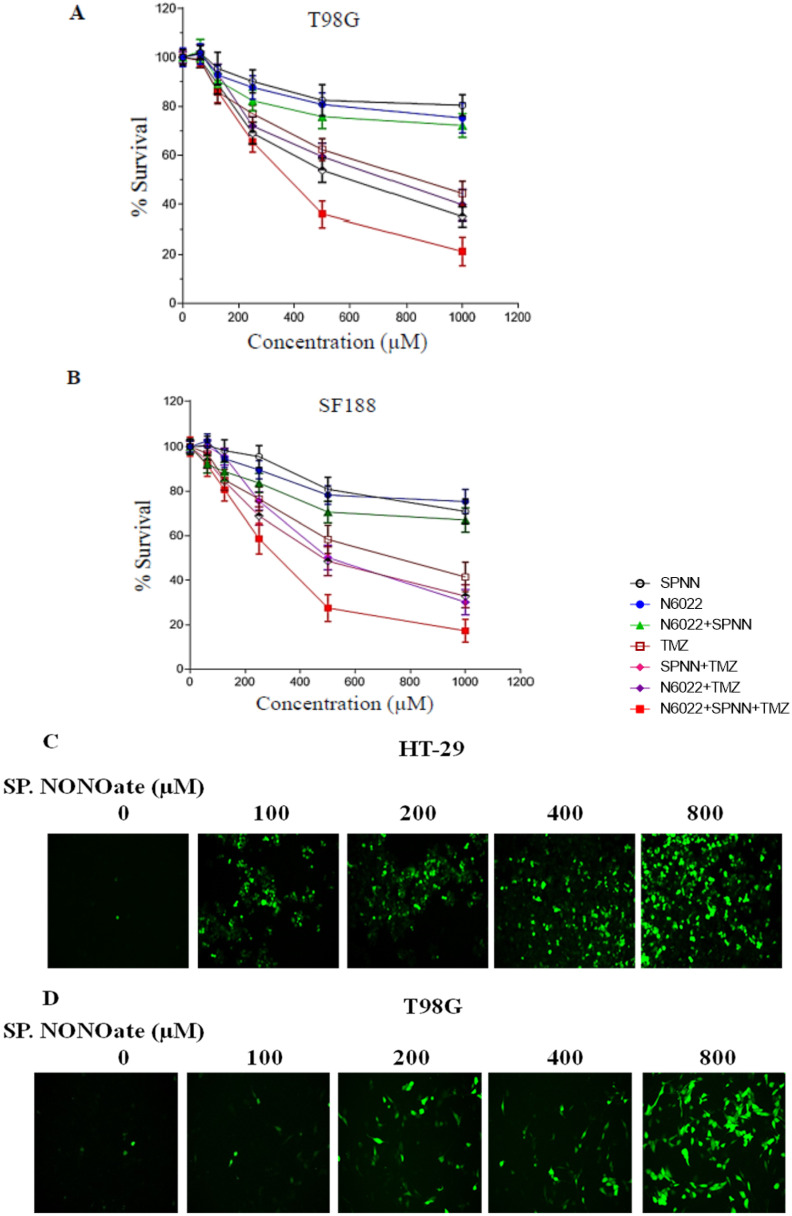

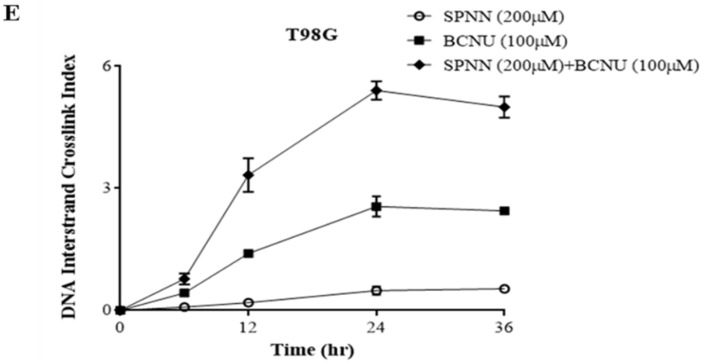
TMZ-mediated cell killing with or without pre-exposure to spermine NONOate and N6022 in (**A**) SF188 and (**B**) T98G GBM cells. Cell growth inhibition was analyzed by the MTT assay. Cells were pre-exposed to either spermine NONOate or N6022 for 24 h and then were treated with different drugs. Spermine NONOate induced ROS elevation in (**C**) HT29 and (**D**) T98G cells as measured by fluorescence intensity associated with DCF-DA. Tumor cells were treated with spermine NONOate for 3 h after the addition of DCF-DA. (**E**) Spermine NONOate increases the alkylation DNA damage induced by MGMT-targeted alkylating agents. Kinetics of DNA interstrand cross-links formed in T98G cells after treatment with 100 μM BCNU with or without spermine NONOate exposure is shown. Cells were treated or untreated with 50 μM spermine NONOate for 12 h to deplete the MGMT protein. They were then exposed to BCNU. At times specified, the cells were harvested, DNA isolated and the extent of interstrand cross-linking of DNA was determined by the ethidium bromide fluorescence assay as described in the methods. Values are mean ± SD. The results were significant at *p* < 0.05.

The suppression of MGMT-mediated DNA repair is conceived to amplify alkylation DNA damage and interstrand cross-linking of DNA induced by bifunctional alkylating agents. The O^6^-chloroethyl guanine residues that are generated by BCNU are converted to GC crosslinks and lack of MGMT increases the crosslink production. We tested this hypothesis by treating the T98G cells with spermine NONOate for 12 h followed by BCNU. To determine the extent of DNA crosslinking, the ethidium bromide fluorescence assay was used. The results showed a ~2.5-fold increase in net interstrand cross-links when cells were treated with a combination of spermine NONOate and BCNU compared to BCNU alone (Figure 5E).

Spermidine NONOate, being a general nitrosylating agent, is expected to have off-target effects influencing multiple processes in cell physiology. Consistent with this postulate, we observed a significant elevation of ROS production in a concentration-dependent manner in HT-29 and T98G cells (Figure 5C,D). Next, we determined the potentiation of temozolomide (TMZ)-induced cell killing by spermidine NONOate. While the nitrosylating agent alone or its combination with N-6022 did not affect the extent of cell-killing, there was a significant decrease in cell survival when the NONOate and the GSNOR inhibitor were combined with TMZ (Figure 5A,B). We calculated a 2-fold increase in TMZ cytotoxicity when the nitrosylator and GSNOR inhibitor were combined.

### 3.4. Evidence That Interaction of hGTX and Spermine NONOate with the Active Site Cysteine of MGMT Leads to DNA Repair Inactivation

**Figure 6:**
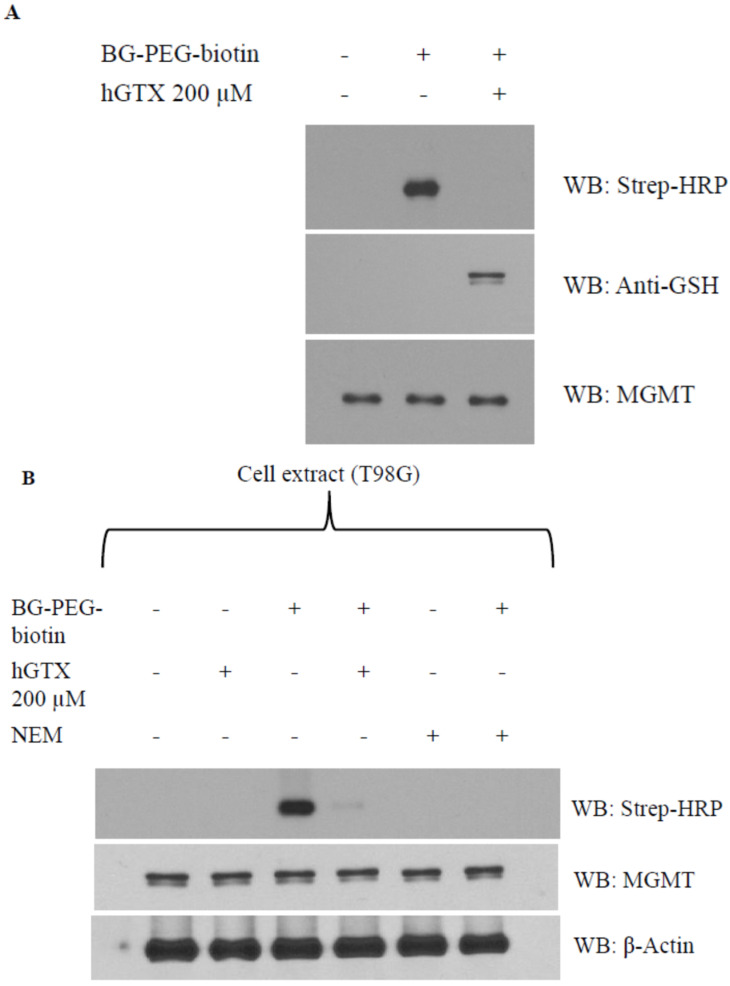
(**A**) Evidence that Cys145 of MGMT is the target site for modification by hGTX. Purified rMGMT protein was incubated with 200 μM hGTX in 40 mM Tris–HCl, pH 8.0, 1 mM EDTA for 20 min at 37 °C. Biotinylated O^6^-benzylguanine (BG–PEG–biotin; 5 μM) was then added and incubations continued for 15 min. The samples were electrophoresed, blotted, and probed with streptavidin–horseradish peroxidase (strep-HRP) to detect the protein-bound biotin (upper panel). The blot was reprobed with antibodies to glutathione and MGMT (lower panels). (**B**) hGTX abrogates the binding of the BG probe to cellular MGMT. T98G cell extracts containing MGMT were incubated with hGTX or N-ethylmaleimide (0.5 mM) for 20 min followed by BG–PEG–biotin. Streptavidin–horseradish peroxidase was used to probe the blot (upper panel). The blot was reprobed with an MGMT antibody (lower panel). (Whole blot See Figure S4).

**Figure 7:**
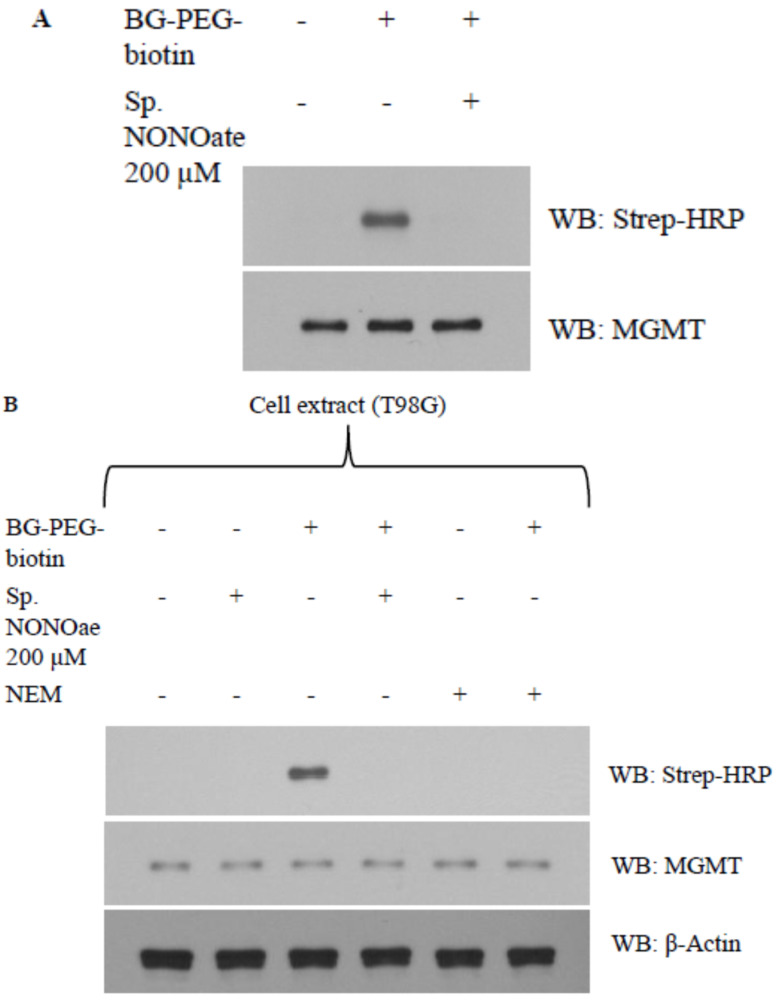
(**A**) Evidence that Cys145 of MGMT is the target site for spermine NONOate. Purified rMGMT protein was incubated with 200 μM spermine NONOate in 40 mM Tris–HCl, pH 8.0, 1 mM EDTA for 20 min at 37 °C. BG–PEG–biotin (5 μM) was then added and incubations continued for 15 min. The samples were electrophoresed, blotted, and probed with streptavidin–horseradish peroxidase to detect the protein-bound biotin (upper panel). The blot was reprobed with antibody to MGMT (lower panel). (**B**) spermine NONOate abrogates the binding of the BG probe to cellular MGMT. T98G cell extracts containing MGMT were incubated with spermine NONOate or N-ethylmaleimide (0.5 mM) for 20 min followed by BG–PEG–biotin. Streptavidin–horseradish peroxidase was used to probe the resulting blot (upper panel). The blot reprobed with MGMT antibody shows equal protein loading (lower panel). (Whole blot See Figure S5).

With cysteine residues in particular, the reactive cysteines present on protein surfaces are primary targets of thiolation and nitrosylation. The Cys145 that accepts the alkyl groups is the most reactive cysteine out of five cysteine residues in the human MGMT protein. To dissect the mechanism of whether interference with cys145 of MGMT results in its inhibition by hGTX and spermine NONOate, we used a biotin-labeled O6-benzylguanine (BG) probe (Covalys Biosciences, Berne, Switzerland), which binds specifically binds this cysteine forming a covalent S-benzyl cysteine [18,19,20,21]. In these experiments, purified recombinant MGMT protein was incubated with hGTX or spermine NONOate followed by BG–PEG–biotin along with controls. The reaction mixtures were subjected to SDS-PAGE followed by electrophoretic transfer to PVDF membranes. The protein-bound biotin was detected by using the streptavidin–horseradish peroxidase on the blots. Pre-exposure of purified MGMT protein with hGTX (Figure 6A) and spermine NONOate (Figure 7A) eliminated the binding of BG-biotin. When the same blots were probed with MGMT antibodies, MGMT protein levels were equal in all lanes confirming the lack of binding of the biotinylated BG. Next, we replaced the rMGMT with endogenous MGMT protein by using tumor cell extracts and performing BG-biotin binding assays. Again, treatment of extracts with hGTX or spermine NONOate before labeling with BG–PEG–biotin resulted in the elimination of Western blot signals (Figure 6B and Figure 7B). N-ethylmalamide used as a control to block the active site cys145 of MGMT also prevented the BG labeling of the protein. In the hGTX-exposed samples, GSH antibodies recognized the glutathionylated MGMT protein (Figure 6A, middle blot). These data confirm that hGTX and spermine NONOate modify the active site cys145 of MGMT, by glutathionylation and nitrosylation, respectively, consequently inactivating its DNA repair function.

### 3.5. MGMT Inhibition by hGTX Enhances and Prolongs the BCNU and TMZ-Induced G2/M Cell Cycle Arrest in GBM Cells

**Figure 8:**
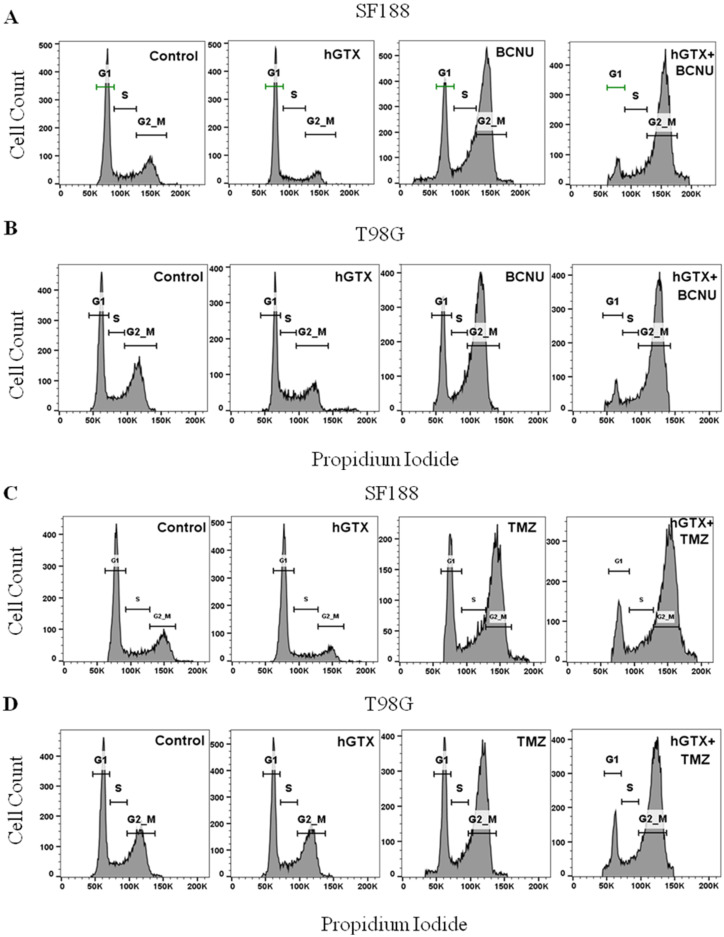
Enhancement of the G2/M blockade induced by BCNU and TMZ by hGTX. Histograms showing results of flow cytometry in SF188 and T98G cells following hGTX, BCNU, TMZ, and combinations of hGTX with the alkylating agents (**A**–**D**) are shown.

Next, we performed cell cycle analysis to determine the consequences of MGMT inactivation by hGTX on changes in cell cycle progression induced by BCNU and TMZ. Two human GBM cells, SF188 and T98G were treated with the alkylating agents alone or in combination with hGTX. The histograms generated from propidium iodide-stained cells showed that 100 μM hGTX alone did not alter cell cycle phase transitions in both cell lines (Figure 8). However, the alkylating agents BCNU and TMZ, as anticipated [39], produced a significant G2/M blockade. Furthermore, the cells pretreated with hGTX and then exposed to BCNU or TMZ exhibited a lower number of cells in the G1 phase along with a greater accumulation of cells in the G2/M phase (Figure 8). Based on the evidence provided so far, it is clear that the nontoxic drug hGTX, acting through the inhibition of MGMT, is capable of potentiating the cytotoxic effects of alkylating agents.

### 3.6. Brief Pretreatment with hGTX Sensitizes MGMT-Proficient Tumor Cells to Alkylating Agents

**Figure 9:**
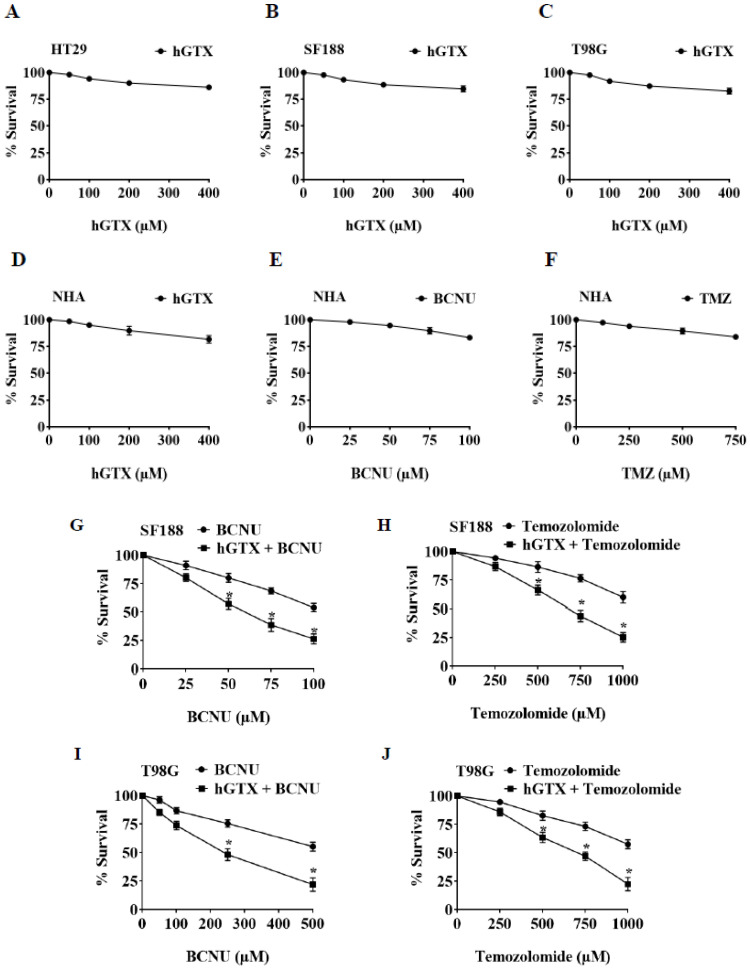
Cytotoxicity of hGTX against MGMT proficient (**A**) HT29, (**B**) SF188, and (**C**) T98G cancer cells. The data show that hGTX alone did not exert significant cytotoxicity. Cell growth inhibition was analyzed with 200 and 400 μΜ for 24 h. In normal human astrocyte culture (NHA) treated with hGTX, BCNU, and TMZ no cytotoxicity was observed (**D**–**F**). However, hGTX pre-exposure sensitized the brain tumor cells to the clinically used alkylating agents. (**G**–**J**). SF188 and T98G cells were preincubated with 100 μΜ hGTX followed by treatment with different agents using MTT assay. Cells were treated with various concentrations of hGTX (50, 100, 200, and 400 μM), BCNU (250–1000 μM), and TMZ (250–1000 μM) for 24 h. The data represent the results of three independent experiments performed in triplicate. Values are mean ± SD. The results presented in panels (**G**–**J**) were significant at *p* < 0.05. *, indicates a statistically significant difference compared to individual agents (BCNU and temozolomide alone).

MTT assays were performed to determine the extent of tumor cell survival following exposure to hGTX. hGTX by itself, over a concentration range of 100–400 μM for 24 h, showed no toxic effects on GBM cell lines. Based on this, we selected 100 μM hGTX preincubation for 24 h to test the potentiation of alkylating agents. In this setting, TMZ (0–1000 μM) + hGTX combination showed a 2- to 3-fold enhanced cytotoxicity compared with TMZ alone. With BCNU similar results were observed (Figure 9). When normal human astrocytes were exposed to hGTX, no significant cytotoxicity was observed (Figure 9D). The potentiation of cytotoxicity by hGTX and spermine NONOate (Figure 5A,B), although moderate under the conditions used, indicates their utility in a clinical setting. Mechanistically, the MGMT inhibition by thiolation and nitrosylation is likely to be critical in alkylator-induced tumor cell killing; however, we agree there will be several off-target effects.

### 3.7. Treatment with Thiolating and Nitrosylating Agents Promote ROS Generation in MGMT-Proficient Cells

**Figure 10:**
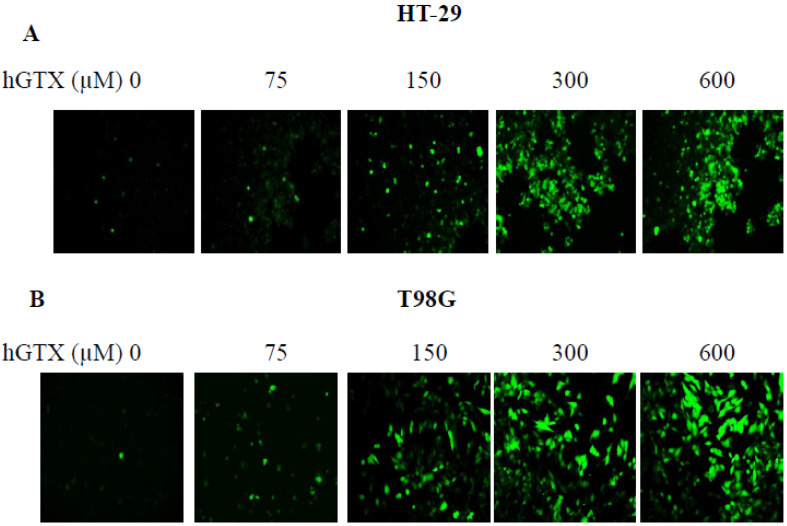
hGTX-induced ROS elevation in HT29 (**A**) and T98G (**B**) cells. ROS levels were measured by fluorescence intensity associated with DCF-DA in the oxidative milieu. Tumor cells were treated with hGTX (75, 150, 300, and 600 μM) for 3 h after the addition of DCF-DA. ROS levels were then measured using a plate reader as described in the methods.

The ability of HGTX to induce oxidative stress in MGMT-proficient tumor cell lines was explored using the redox probe DCF-DA. DCF-DA oxidizes rapidly to a highly fluorescent 2′, 7′- Dichlorodihydrofluorescein (DCF) in stressed cells, and the fluorescence intensity is proportional to the cytosolic ROS levels. The increase in ROS levels was confirmed when cells were incubated with DCF-DA for 30 min, followed by treatment with hGTX (Figure 10A,B) and spermine NONOate (Figure 5C,D). However, there was no significant difference in ROS levels in normal human astrocytes after hGTX treatment for the same period indicating that ROS production was selective for cancer cells, but not for normal epithelial cells.

### 3.8. Evidence That GSNOR Inhibition Prolongs the MGMT Deficient State

**Figure 11:**
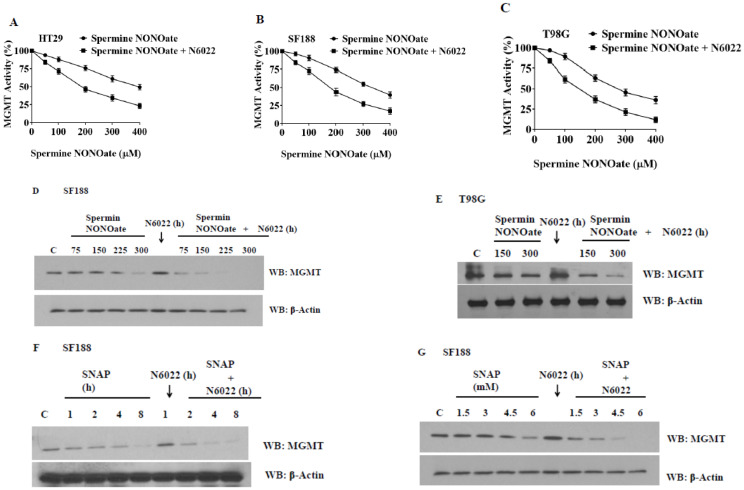
Inhibition of S-nitrosoglutathione reductase (GSNOR) activity results in increased nitrosylation of MGMT by spermine NONOate, and greater inhibition/depletion of the DNA repair protein. Cell extracts were assayed for MGMT activity in panels (**A**) HT29, (**B**) SF188, and (**C**) T98G. Panels (**D**,**E**) show the MGMT protein levels in the same experiments. In (**F**,**G**), cells were exposed to SNAP (S-nitroso-L-penicillamine), another representative nitrosylating agent. An augmented decrease in MGMT protein when the nitrosylating agents were combined with N6022 is evident. (Whole blot See Figure S9).

We investigated whether GSNOR inhibition would be able to augment the nitrosylation of MGMT and increase its rate of degradation. For this, we used N6022, a specific GSNOR inhibitor available commercially. N6022 was used either alone or in combination with spermine NONOate. First, the combination was tested on MGMT activity. A greater level of MGMT inactivation was evident in the combination (Figure 11A–C). In the next experiments, we preincubated the GBM cells with N6022 for 1 h and then added the nitrosylating agent. Western blotting was performed to assess the MGMT protein levels. It was found that N6022 by itself was unable to inhibit the MGMT protein. However, when NO-donating agents were added, N6022 was able to extend and enhance the levels of MGMT breakdown (Figure 11D,E) With SNAP (S-nitrosopenicillamine) and spermine NONOate, MGMT elimination was around 80–90%. Spermine NONOate generated a similar inhibition pattern in both SF188 and T98G cells. On the other hand, when we used spermine (a component of spermine NONOate) alone, MGMT levels remained similar to the control suggesting that GSNOR inhibition resulted in an extended retention of the nitrosylated MGMT, which was then degraded (Figure 11F,G). These new approaches to targeting MGMT nitrosylation bear clinical significance.

### 3.9. Potentiation of Antitumor Efficacy of Alkylating Agents by Thiolating and Nitrosylating Agents in Xenograft Settings

**Figure 12:**
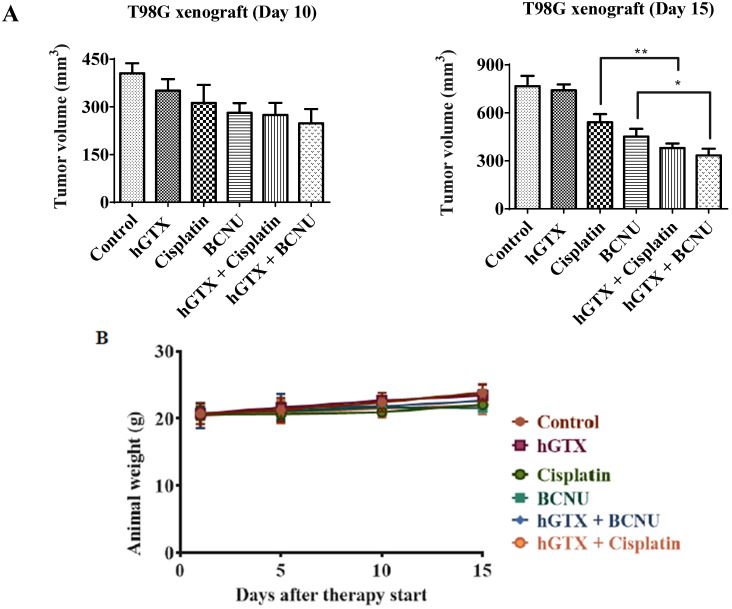

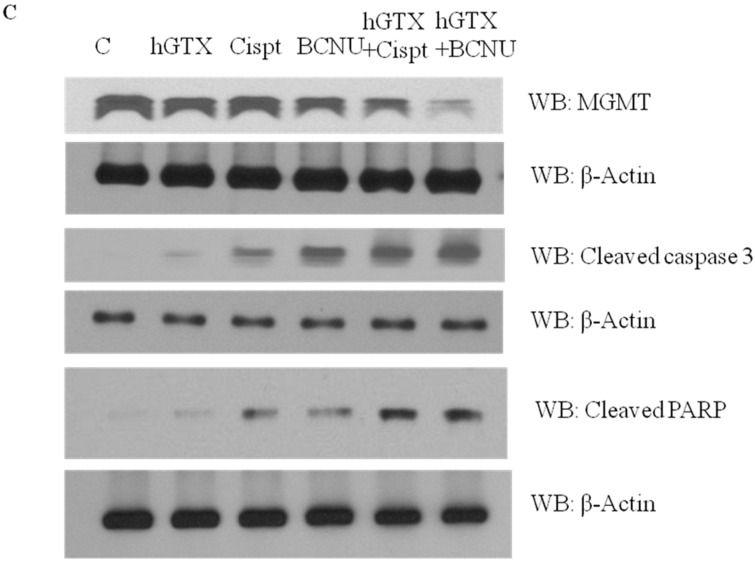
(**A**) Bar diagrams representing enhanced regression of T98G tumors in hGTX + cisplatin and hGTX + BCNU groups. Tumor volumes shown are mean ± SD. One-way ANOVA followed by Tukey’s post hoc analysis was used to determine significant differences among the groups. These analyses showed a greater decrease in tumor burden in combination of hGTX with cisplatin and BCNU compared to the individual agents alone. * denotes significant difference, * *p* < 0.05, ** *p* < 0.01. (**B**) After 15 days of treatment, there was no difference in the body weights between the vehicle and treatment groups. (**C**) Altered levels of MGMT and apoptotic regulatory proteins in T98G tumor lysates from xenografts. (Whole blot See Figure S6).

To validate our findings of MGMT inhibition by hGTX and nitrosylating compounds in preclinical settings, we developed subcutaneous xenografts by injecting many MGMT proficient cell lines in female nu/nu mice and determined the antitumor efficacy and MGMT activity and protein levels in clarified tumor lysates. In the first study with the T98G xenograft, significant tumor regression was observed after treatment with cisplatin and BCNU. However, when the mice were pretreated with hGTX, tumor growth inhibition increased by 2-fold (Figure 12A). No changes were discernible in the body weight of animals indicating a lack of toxicity (Figure 12B). The morphology of the normal tissues as assessed by H&E staining in the mice bearing tumors did not show alterations (Figure 15). When the lysates from the excised tumors were immunoblotted, there was an apparent decrease in MGMT protein levels; this decrease was accompanied by enhanced levels of both the cleaved PARP and cleaved caspase-3 in tumors verifying that hGTX along with the alkylating agents triggered apoptotic cascades in tumor tissues (Figure 12C). Further, the MGMT activity in tumor lysates was also diminished considerably after treatment with the combinations of hGTX and cisplatin, hGTX + BCNU in T98G xenografts and hGTX + TMZ, NCX + BCNU, and NCX + TMZ combinations in the HT-29-luc2 (Figure 14), reflecting the greater alkylation DNA damage incurred in vivo.

**Figure 13:**
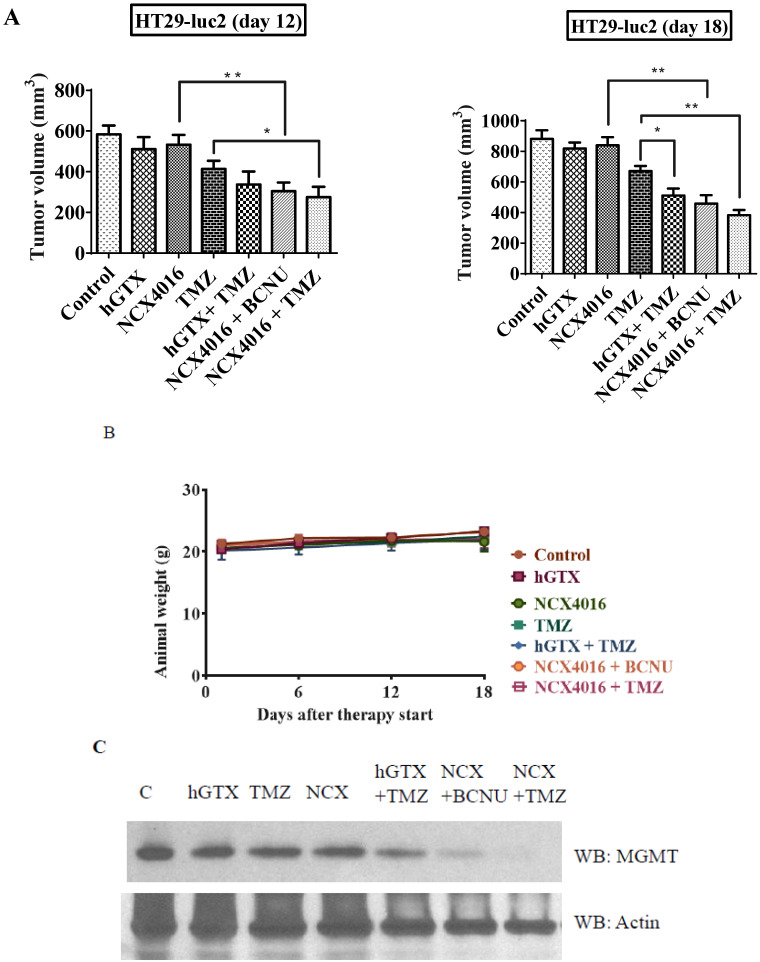

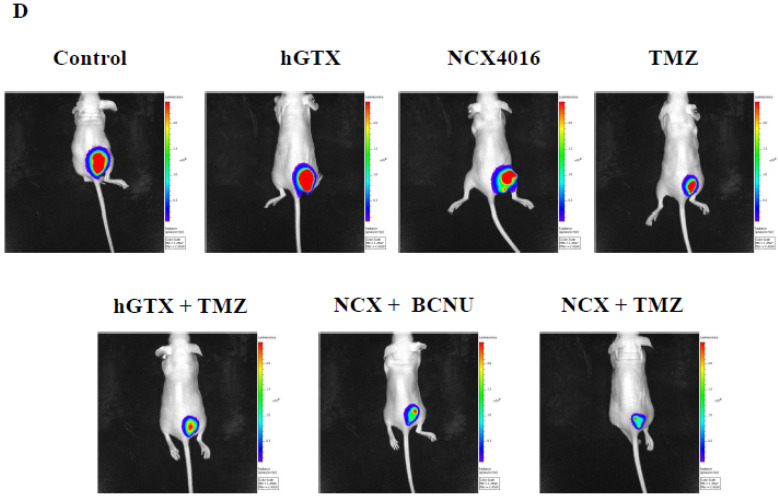
(**A**) Bar diagrams represent enhanced regression of HT29-luc2 subcutaneous tumors by hGTX or nitroaspirin (NCX4016) and their combinations with the alkylating agents (TMZ, BCNU). Tumor volumes shown are mean ± SD. One-way ANOVA followed by Tukey’s post hoc analysis was used to determine the significant differences among the groups. * denotes significant difference, * *p* < 0.05, ** *p* < 0.01. (**B**) The treatment regimens did not demonstrate statistically significant weight differences compared to the control. (**C**) The combination groups showed a decrease in MGMT protein as revealed by the immunoblot analysis. (**D**) Representative images of mice from the drug-treated groups also showed decreased bioluminescence at the tumor sites. (Whole blot See Figure S7).

In the second study using the HT29-luc2 xenografts, tumor growth delay was evident in the mice treated with combinations of hGTX or nitroaspirin (NCX4016) with TMZ and BCNU. MGMT protein levels were downregulated in the hGTX + TMZ, NCX + BCNU, and NCX + TMZ combination groups (Figure 13C) and correlated well with the increased drug efficacy. As shown in Figure 13D, mice treated with combination regimens exhibited diminished bioluminescence compared to the TMZ-alone groups. Consistent with our previous findings that NCX-4016 inactivates MGMT [35,36], a significant increase in TMZ efficacy was observed. The drug efficacy in these experiments was also quantitated by conventional tumor volume measurements (using Vernier calipers), and these data are shown in bar graphs (Figure 13A). In the third study using T98G xenografts, there was a greater and significant decrease in tumor volumes in the mice treated with combinations of spermine NONOate and N6022 with TMZ (Figure 16A,B). Neither of these groups exhibited significant alterations in body weight (Figure 16C). A decrease in MGMT protein levels as well as a raise in cleaved PARP levels was also evident in the tumor lysates from these experiments (Figure 16D). Collectively, these data agree with and confirm the second arm of our study focusing on nitrosylation of MGMT and extended retention of nitrosylated DNA repair protein to obtain improved antitumor efficacy.

**Figure 14:**
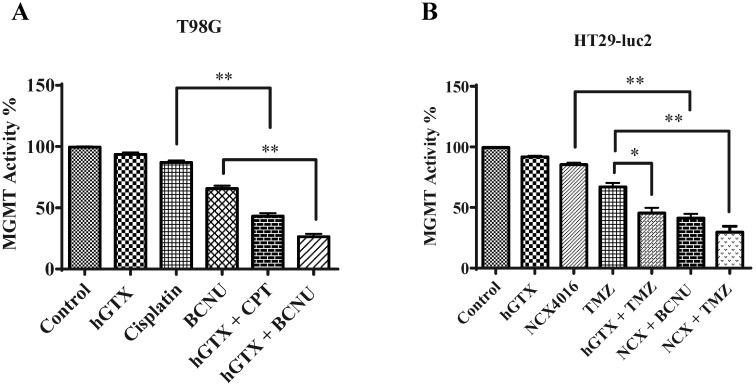
Effect of combination treatments of hGTX and NCX4016 with alkylating agents on MGMT activity levels in tumor tissues from T98G (**A**) and HT29-luc2 (**B**) xenografts. After drug treatments (*n* = 8), tumors were excised, crude extracts were prepared followed by assay of MGMT activity in triplicate. A greater depletion of MGMT activity resulted when hGTX was combined with alkylating agents. One-way ANOVA followed by Tukey’s post hoc analysis was used to determine the significant differences among the groups. * denotes significant difference, * *p* < 0.05, ** *p* < 0.01.

**Figure 15:**
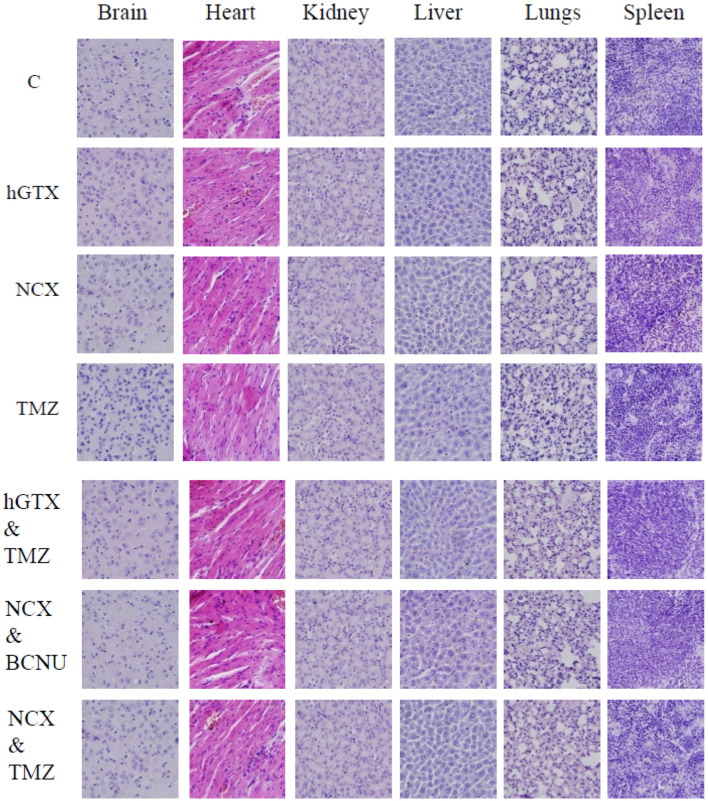
H&E staining of major organ sections from nude mice administered hGTX, NCX4016, and anticancer drugs. Scale bar: 100 μm. The tissue morphology was similar in different groups indicating the absence of discernible toxicity.

**Figure 16:**
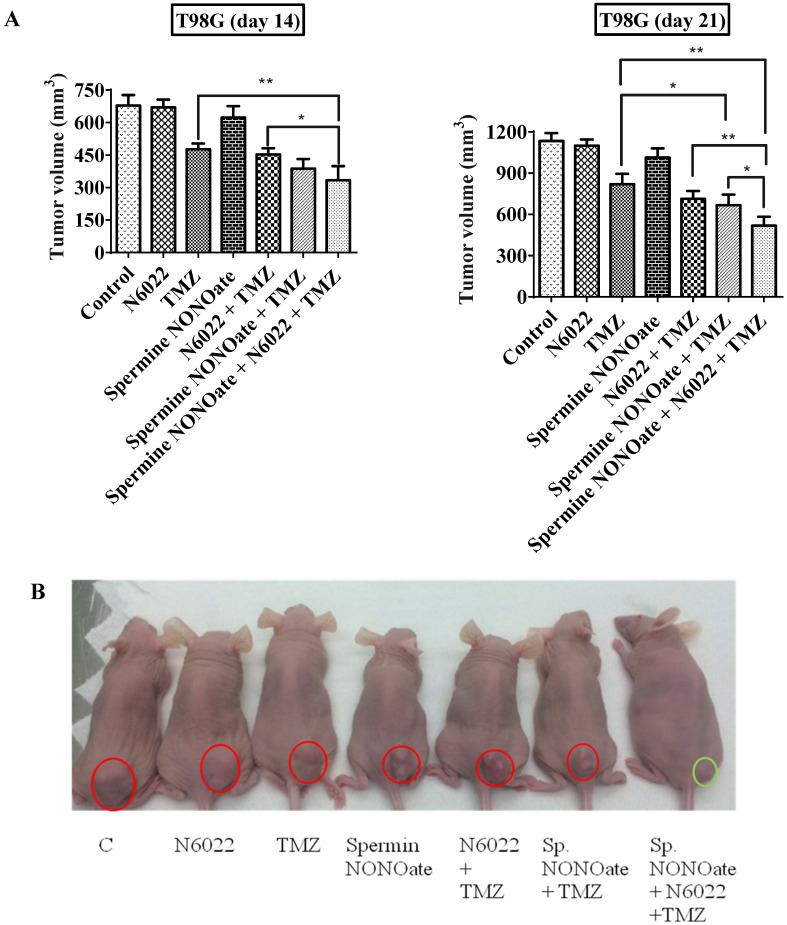

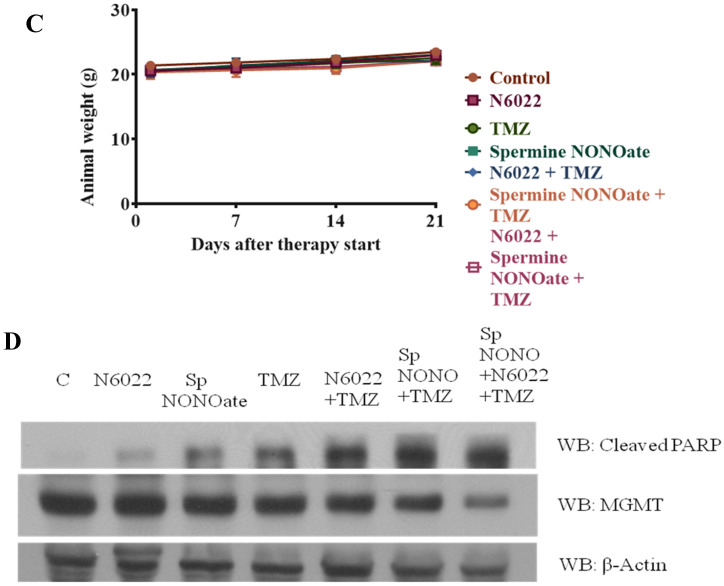
(**A**) Bar diagrams represent the potentiation of tumor regression in T98G subcutaneous xenografts by spermine NONOate, N6022 in combination with temozolimide (TMZ). Drug treatments were initiated when the tumor volumes reached 100 mm^3^. Spermine NONOate was administered 0.4 mg by intra-tumoral injections twice a week. The other drugs were given intraperitoneally as described in the methods. Tumor volumes shown are mean ± SD. One-way ANOVA followed by Tukey’s post hoc analysis was used to determine the significant difference among different groups. * denotes significant difference, * *p* < 0.05, ** *p* < 0.01. (**B**) Representative images of mice from the drug treatment groups. Tumor locations in mice are circled in red and their absence in green. (**C**) The treatment regimens did not induce statistically significant weight differences compared to the control. (**D**) A decrease in MGMT protein and an increase in cleaved PARP was observed by Western blot analyses of tumor lysates in the combination groups. (Whole blot See Figure S8).

**Figure 17:**
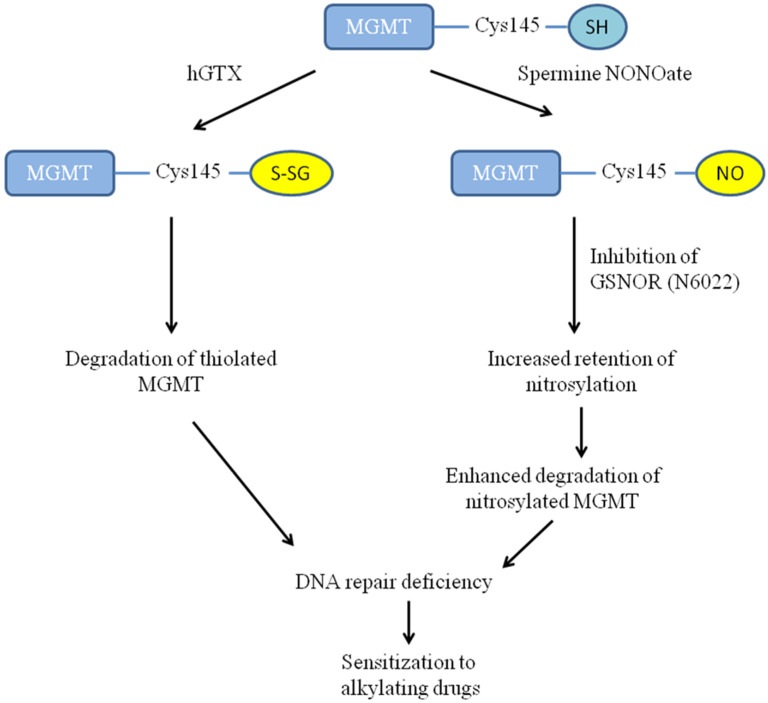
Proposed scheme of the consequences of MGMT inhibition by hGTX and spermine NONOate and increased tumor sensitivity to alkylating agents.

Overall, as represented in Figure 17, our data obtained from cell culture and animal studies in this report establish that the DNA repair capacity of MGMT can be curtailed by thiolation and nitrosylation events which in turn can be exploited for improving the brain tumor therapy.

## 4. Discussion

This study addressed new ways of inhibiting human MGMT targeting the biochemical reactivity of its active site cysteine residue. We induced mild oxidative and nitrosative stresses using agents that mimicked endogenous redox components. The stresses, albeit pleiotropic with the potential for affecting many redox regulatory processes and signaling, did trigger significant inhibition of MGMT in brain tumor cells in cell culture and xenograft settings; no toxicity on host tissues was observed. The DNA repair inhibition by the agents did not induce cytotoxicity by themselves but effectively increased the cell killing in combination with alkylators both in cell culture and tumor xenograft settings.

The mixed disulfide formation between reactive cysteines in proteins with either cysteine (thiolation) or glutathione (glutathionylation) is a critical posttranslational modification that occurs in cells with oxidative stress; cells exposed to NOV-002 and hGTX respond with higher levels of glutathionylation in response to redox imbalance (higher GSSG: GSH ratios) [29,30]. Glutathionylation has largely been perceived as a protective mechanism against the irreversible oxidation of -SH groups in redox-sensitive proteins, often at the expense of temporary loss of their activities [40,41]. However, the cells are likely to recognize the thiolated and cysteine oxidized proteins as inactive and trigger their degradation [37]. Very much similar to phosphorylation, thiolation has received attention for drug discovery but the potential of thiol modifications as a therapeutic strategy is still unexplored [42,43]. hGTX demonstrated significant and irreversible inhibition of MGMT protein, both in cell culture and animal models. Several reports have also demonstrated MGMT inhibition by nitrosylation [20,44,45]. Therefore, we explored spermine NONOate as a model nitrosylating compound for inhibiting the MGMT and potentiating the GBM cytotoxicity by alkylating agents. A major finding of this study is the demonstration that MGMT inhibition through these redox-sensitive protein modifications occurs fairly rapidly to create an MGMT-deficient state, just similar to that engineered by O^6^-benzylguanine in tumor cells.

Previously, researchers have used NO donors such as PABA/NO and DEA/NO for inducing chemosensitivity in glioma cells [46]. These NO donors are non-specific and have several undesirable effects. Since higher doses were needed for therapeutic effect, their clinical use is rather restricted [47,48]. In this regard, spermine NONOate appears to be a better alternative because it was not toxic in vitro and in vivo. Nitrosylation-induced MGMT deficiency was extended by the inhibition of S-nitrosoglutathione reductase (GSNOR). The retention of nitrosylated MGMT in cells resulted in a greater degradation of the protein. The formation of S-glutathionylcysteine and S-nitrosylcysteine at Cys-145, and possibly nitrosylation of Tyr114 of MGMT likely enable efficient ubiquitination and subsequent proteasomal digestion of MGMT. The observation made by Wei et al. in GSNOR null mice that hepatic MGMT protein is deficient [49] is supportive of our findings and consistent with the S-nitrosylation of MGMT as a physiological mechanism.

It is estimated ninety thousand new cases of primary brain tumors are diagnosed every year in the United States [50]. Among pediatric patients, brain tumors are the second leading cause of cancer deaths just next to leukemia. Chemotherapy using alkylating agents remains a mainstay in brain tumor therapy; however, the outcomes have remained dismal for malignant gliomas. There is an urgent need for fresh, minimally toxic tumor-selective cytotoxic drugs, not only for cure, but also to synergize with radiation, and often to use alone to delay or avoid the neurotoxic effects of irradiation. Compared to the MGMT pseudosubstrate such as the O6-benzylguanine to which tumors tend to develop resistance and result in therapy failure, the redox-driven approaches, which act through multiple mechanisms, are unlikely to induce resistance. Further, any toxicities due to thiolating and nitrosylating agents may be ameliorated through glutathione augmentation in tissues by using cysteine prodrugs, such as 2-oxothiazolidine-4-carboxylic acid (OTC) [51,52] or glutathione esters [53]. Taken together, our findings support the theme of inducing an MGMT deficient state through redox-altering agents for improving brain tumor treatment.

## Data Availability

The raw data to create figures are available upon request.

## Supplementary Materials

The following supporting information can be downloaded at: https://www.mdpi.com/article/10.3390/diseases13020032/s1, Figures S1–S9.

## References

[1] Pegg A.E. (2000). Repair of O6-alkylguanine by alkyltransferases. Mutat. Res..

[2] Mishina Y., Duguid E.M., He C. (2006). Direct reversal of DNA alkylation damage. Chem. Rev..

[3] Margison G.P., Santibáñez Koref M.F., Povey A.C. (2002). Mechanisms of carcinogenicity/chemotherapy by O6-methylguanine. Mutagenesis.

[4] Srivenugopal K.S., Yuan X.H., Friedman H.S., Ali-Osman F. (1996). Ubiquitination-dependent proteolysis of O6-methylguanine-DNA methyltransferase in human and murine tumor cells following inactivation with O6-benzylguanine or 1,3-bis(2-chloroethyl)-1-nitrosourea. Biochemistry.

[5] Gerson S.L. (2002). Clinical relevance of MGMT in the treatment of cancer. J. Clin. Oncol..

[6] Kaina B., Margison G.P., Christmann M. (2010). Targeting O6-methylguanine-DNA methyltransferase with specific inhibitors as a strategy in cancer therapy. Cell. Mol. Life Sci..

[7] Rabik C.A., Njoku M.C., Dolan M.E. (2006). Inactivation of O6-alkylguanine DNA alkyltransferase as a means to enhance chemotherapy. Cancer Treat. Rev..

[8] Kreklau E.L., Kurpad C., Williams D.A., Erickson L.C. (1999). Prolonged inhibition of O(6)-methylguanine DNA methyltransferase in human tumor cells by O(6)-benzylguanine in vitro and in vivo. J. Pharmacol. Exp. Ther..

[9] Chinnasamy D., Fairbairn L.J., Neuenfeldt J., Treisman J.S., Hanson J.P., Margison G.P., Chinnasamy N. (2004). Lentivirus-mediated expression of mutant MGMT^P140K^ protects human CD34+ cells against the combined toxicity of O6-benzylguanine and 1,3-bis(2-chloroethyl)-nitrosourea or temozolomide. Hum. Gene Ther..

[10] Pegg A.E., Wiest L., Mummert C., Stine L., Moschel R.C., Dolan M.E. (1991). Use of antibodies to human O6-alkylguanine-DNA alkyltransferase to study the content of this protein in cells treated with O6-benzylguanine or N-methyl-N_-nitro-Nnitrosoguanidine. Carcinogenesis.

[11] Kanugula S., Goodtzova K., Pegg A.E. (1998). Probing of conformational changes in human O6-alkylguanine-DNA alkyltransferase protein in its alkylated and DNA bound states by limited proteolysis. Biochem. J..

[12] Xu-Welliver M., Pegg A.E. (2002). Degradation of alkylated form of the DNA repair protein, O6-alkylguanine-DNA alkyltransferase. Carcinogenesis.

[13] Niture S.K., Velu C.S., Bailey N.I., Srivenugopal K.S. (2005). Phosphorylated MGMT in human tumors is insensitive to O6-benzylguanine: Identification of the conserved Tyr114 as a phosphorylation site. Proc. Am. Assoc. Cancer Res..

[14] Velu C.S., Niture S.K., Bailey N.I., Srivenugopal K.S. (2004). Posttranslational regulation of human MGMT by sumoylation in brain tumor cells. Proc. Am. Assoc. Cancer Res..

[15] Srivenugopal K.S., Rawat A., Niture S.K., Paranjpe A., Velu C., Venugopal S.N., Madala H.R., Basak D., Punganuru S.R. (2016). Posttranslational Regulation of O(6)-Methylguanine-DNA Methyltransferase (MGMT) and New Opportunities for Treatment of Brain Cancers. Mini Rev. Med. Chem..

[16] Srivenugopal K.S., Mullapudi S.R.S., Shou J., Ali-Osman F. (2001). Mg^2+^ and ATP-dependent degradation of recombinant O6-methylguanine-DNA methyltransferase protein in human tumor cell extracts. Proc. Am. Assoc. Cancer Res..

[17] Velu C.S., Niture S.K., Bailey N.I., Srivenugopal K.S. (2005). MGMT protein turnover in human tumors is mediated by the ubiquitinproteasome pathway: Phosphorylation-dependent ub-conjugation by the Skp2-SCF complex. Proc. Am. Assoc. Cancer Res..

[18] Guengerich F.P., Fang Q., Liu L., Hachey D.L., Pegg A.E. (2003). O6-alkylguanine-DNA alkyltransferase: Low pKa and high reactivity of cysteine 145. Biochemistry.

[19] Niture S.K., Velu C.S., Bailey N., Srivenugopal K.S. (2004). Human MGMT is a prime target for inactivation by oxidative stress, mediated by glutathionylation and oxidation of the active site cysteine145. Proc. Am. Assoc. Cancer Res..

[20] Liu L., Xu-Welliver M., Kanugula S., Pegg A.E. (2002). Inactivation and degradation of O(6)-alkylguanine-DNA alkyltransferase after reaction with nitric oxide. Cancer Res..

[21] Green L.S., Chun L.E., Patton A.K., Sun X., Rosenthal G.J., Richards J.P. (2012). Mechanism of inhibition for N6022, a first-in-class drug targeting S-nitrosoglutathione reductase. Biochemistry.

[22] Moran J.F., Iturbe-Ormaetxe I., Matamoros M.A., Rubio M.C., Clemente M.R., Brewin N.J., Becana M. (2000). Glutathione and homoglutathione synthetases of legume nodules. Cloning, expression, and subcellular localization. Plant Physiol..

[23] Srivenugopal K.S., Mullapudi S.R., Shou J., Hazra T.K., Ali-Osman F. (2000). Protein phosphorylation is a regulatory mechanism for O6-alkylguanine-DNA alkyltransferase in human brain tumor cells. Cancer Res..

[24] Myrnes B., Norstrand K., Giercksky K.E., Sjunneskog C., Krokan H. (1984). A simplified assay for O6-methylguanine-DNA methyltransferase activity and its application to human neoplastic and nonneoplastic tissues. Carcinogenesis.

[25] Punganuru S.R., Madala H.R., Mikelis C.M., Dixit A., Arutla V., Srivenugopal K.S. (2018). Conception, synthesis, and characterization of a rofecoxib-combretastatin hybrid drug with potent cyclooxygenase-2 (COX-2) inhibiting and microtubule disrupting activities in colon cancer cell culture and xenograft models. Oncotarget.

[26] Ali-Osman F., Rairkar A., Young P. (1995). Formation and repair of 1,3-bis-(2-chloroethyl)-1-nitrosourea and cisplatin induced total genomic DNA interstrand crosslinks in human glioma cells. Cancer Biochem. Biophys..

[27] Madala H.R., Punganuru S.R., Ali-Osman F., Zhang R., Srivenugopal K.S. (2018). Brain- and brain tumor-penetrating disulfiram nanoparticles: Sequence of cytotoxic events and efficacy in human glioma cell lines and intracranial xenografts. Oncotarget.

[28] Punganuru S.R., Madala H.R., Arutla V., Zhang R., Srivenugopal K.S. (2019). Characterization of a highly specific NQO1-activated near-infrared fluorescent probe and its application for in vivo tumor imaging. Sci. Rep..

[29] Townsend D.M., He L., Hutchens S., Garrett T.E., Pazoles C.J., Tew K.D. (2008). NOV-002, a Glutathione disulfide mimetic, as a modulator of cellular redox balance. Cancer Res..

[30] Sara J., Lin H., Garrett T., Tew K.D., Townsend D.M. (2010). Protective effects of a glutathione disulfide mimetic (NOV-002) against cisplatin induced kidney toxicity. Biomed. Pharmacother..

[31] Gumireddy K., Li A., Cao L., Yan J., Liu L., Xu X., Pazoles C., Huang Q. (2013). NOV-002, A glutathione disulfide mimetic, suppresses tumor cell invasion and metastasis. J. Carcinog. Mutagen..

[32] Montero A.J., Diaz-Montero C.M., Deutsch Y.E., Hurley J., Koniaris L.G., Rumboldt T., Yasir S., Jorda M., Garret-Mayer E., Avisar E. (2012). Phase 2 study of neoadjuvant treatment with NOV-002 in combination with doxorubicin and cyclophosphamide followed by docetaxel in patients with HER-2 negative clinical stage II-IIIc breast cancer. Breast Cancer Res. Treat..

[33] Miller M.R., Megson L. (2007). Recent developments in nitric oxide donor drugs. Br. J. Pharmacol..

[34] Corazzi T., Leone M., Maucci R., Corazzi L., Gresele P. (2005). Direct and irreversible inhibition of cyclooxygenase-1 by nitroaspirin (NCX 4016). J Pharmacol Exp Ther..

[35] Paranjpe A., Srivenugopal K.S. (2012). Discovery and characterization of nitroaspirin (NCX-4016) as a powerful and clinically relevant inhibitor of human MGMT for increasing the efficacy of alkylating agents. Cancer Res..

[36] Srivenugopal K.S., Paranjpe A. (2013). The NO-releasing aspirin inactivates and degrades human MGMT more efficiently than O6-benzylguanine and greatly sensitizes brain tumor cells to alkylating agents. Cancer Res..

[37] Paranjpe A., Srivenugopal K.S. (2013). Degradation of NF-κB, p53 and other regulatory redox-sensitive proteins by thiol-conjugating and -nitrosylating drugs in human tumor cells. Carcinogenesis.

[38] Barnett S.D., Buxton I.L.O. (2017). The role of S-nitrosoglutathione reductase (GSNOR) in human disease and therapy. Crit. Rev. Biochem. Mol. Biol..

[39] Yan L., Donze J.R., Liu L. (2005). Inactivated MGMT by O6-benzylguanine is associated with prolonged G2/M arrest in cancer cells treated with BCNU. Oncogene.

[40] Klatt P., Lamas S. (2000). Regulation of protein function by S-glutathiolation in response to oxidative and nitrosative stress. Eur. J. Biochem..

[41] Ghezzi P., Bonetto V., Fratelli M. (2005). Thiol-disulfide balance: From the concept of oxidative stress to that of redox regulation. Antioxid. Redox Signal.

[42] Thomas J.A., Poland B., Honzatko R. (1995). Protein sulfhydryls and their role in the antioxidant function of protein S-thiolation. Arch. Biochem. Biophys..

[43] Giustarini D., Rossi R., Milzani A., Colombo R., Dalle-Donne I. (2004). S-glutathionylation: From redox regulation of protein functions to human diseases. J. Cell Mol. Med..

[44] Laval F., Wink D.A. (1994). Inhibition by nitric oxide of the repair protein, O6-methylguanine-DNA methyltransferase. Carcinogenesis.

[45] Liu L., Xu-Welliver M., Pegg A.E. (2001). Inactivation of DNA repair protein O6-alkylguanine-DNA alkyltransferase by reaction with nitric oxide. FASEB J..

[46] Kurimoto M., Endo S., Hirashima Y., Hamada H., Ogiichi T., Takaku A. (1999). Growth inhibition and radiosensitization of cultured glioma cells by nitric oxide generating agents. J. Neurooncol..

[47] Safdar S., Payne C.A., Tu N.H., Taite L.J. (2012). Targeted nitric oxide delivery preferentially induces glioma cell chemosensitivity via Altered p53 and O6-methylguanine-DNA Methyltransferase activity. Biotechnol. Bioeng..

[48] Kogias E., Osterberg N., Baumer B., Psarras N., Koentges C., Papazoglou A., Saavedra J.E., Keefer L.K., Weyerbrock A. (2012). Growth-inhibitory and chemosensitizing effects of the glutathione-S-transferase-π-activated nitric oxide donor PABA/NO in malignant gliomas. Int. J. Cancer.

[49] Wei W., Li B., Hanes M.A., Kakar S., Chen X., Liu L. (2010). S-nitrosylation from GSNOR Deficiency Impairs DNA Repair and Promotes Hepatocarcinogenesis. Sci. Transl. Med..

[50] American Brain Tumor Association: Brain Tumor Statistics. http://www.abta.org/about-us/news/brain-tumor-statistics/.

[51] Williamson J.M., Meister A. (1981). Stimulation of hepatic glutathione formation by administration of L-2-oxothiazolidine-4-carboxylate, a 5-oxo-L-prolinase substrate. Proc. Natl. Acad. Sci. USA.

[52] Williamson J.M., Boettcher B., Meister A. (1982). Intracellular cysteine delivery system that protects against toxicity by promoting glutathione synthesis. Proc. Natl. Acad. Sci. USA.

[53] Teicher B.A., Crawford J.M., Holden S.A., Lin Y., Cathcart K.N., Luchette C.A., Flatow J. (1988). Glutathione monoethyl ester can selectively protect liver from high dose BCNU or cyclophosphamide. Cancer.

