# Supplementary material for: Novel Pathways of Oxidative and Nitrosative Inactivation of the Human MGMT Protein in Colon Cancer and Glioblastoma Cells: Increased Efficacy of Alkylating Agents In Vitro and In Vivo
Source: Diseases. 2025 Jan 25;13(2):32. doi: 10.3390/diseases13020032 (PMC11854478; doi:10.3390/diseases13020032)

Supplementary data

Basak D et al.

X-ray film images of western blots.

Figure numbers correspond to the designations in the manuscript

Figure S1. Whole blot of Figure 2.

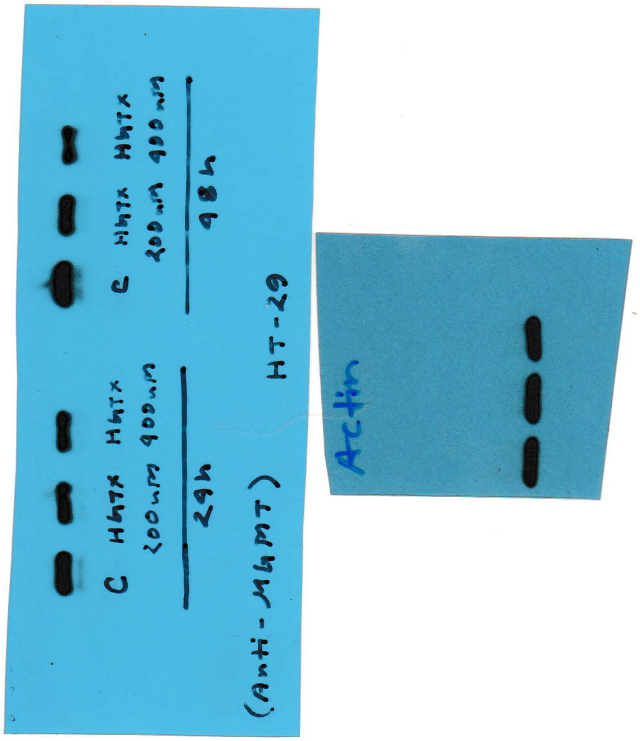

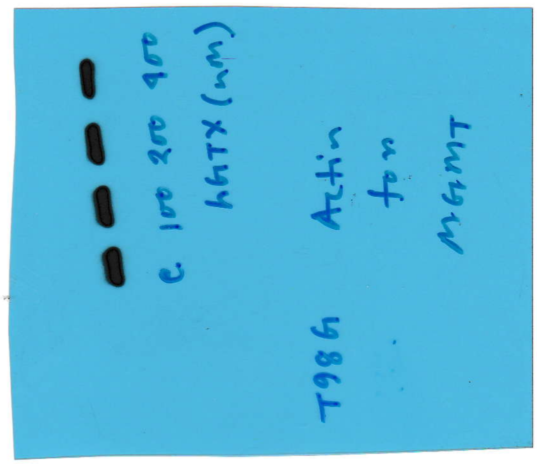

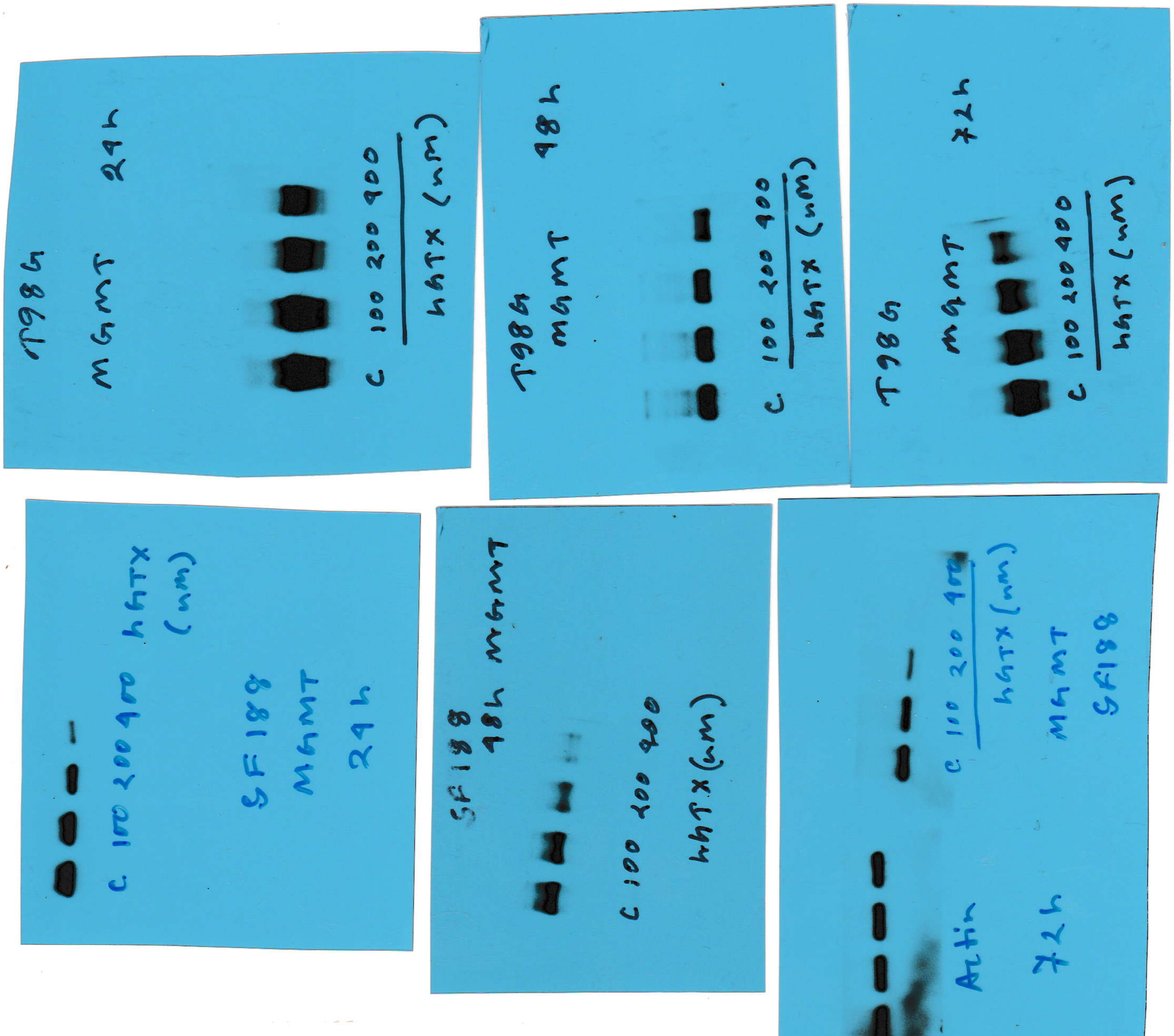

Figure S2. Whole blot of Figure 3.

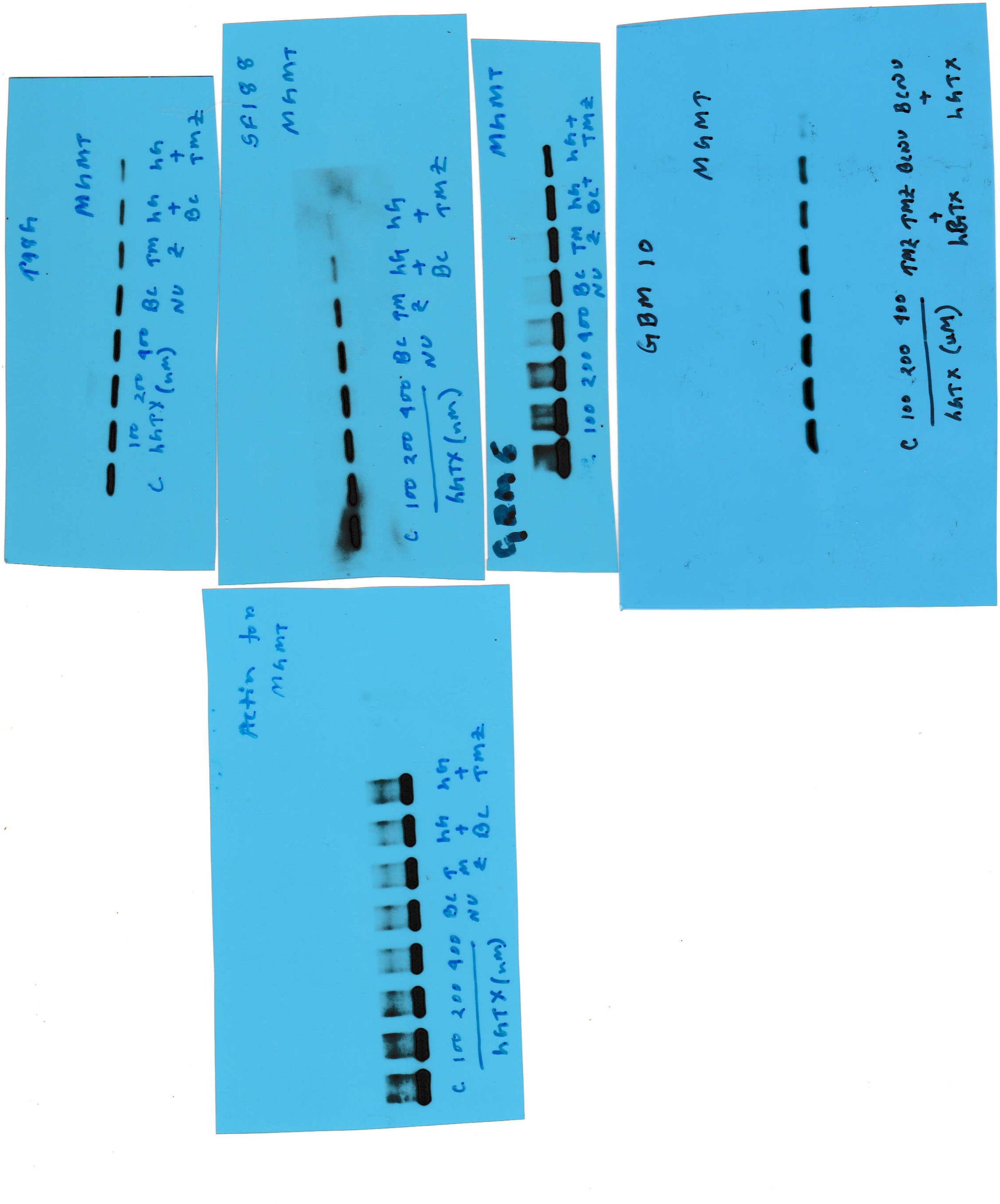

Figure S3. Whole blot of Figure 4 EF

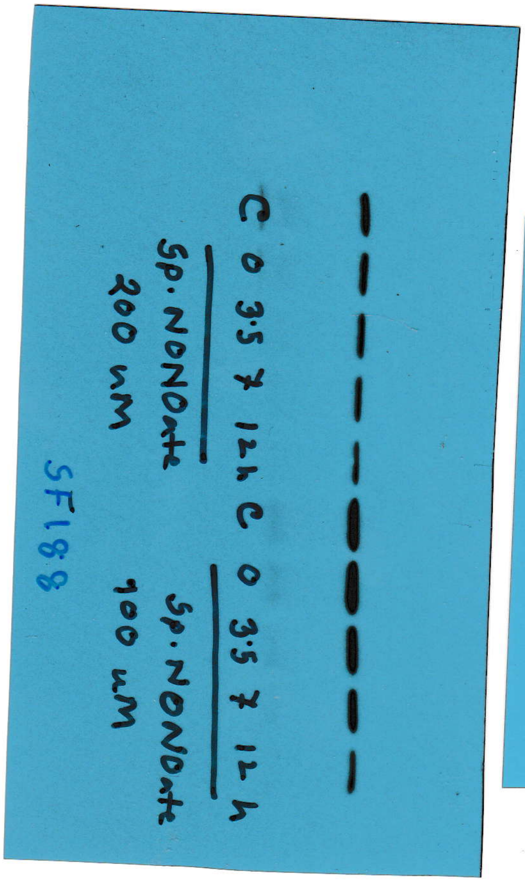

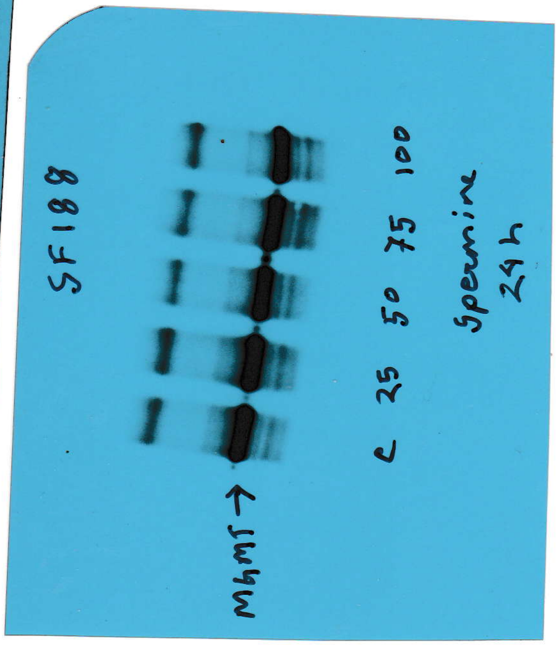

Figure S4. Whole blot of Figure 6AB

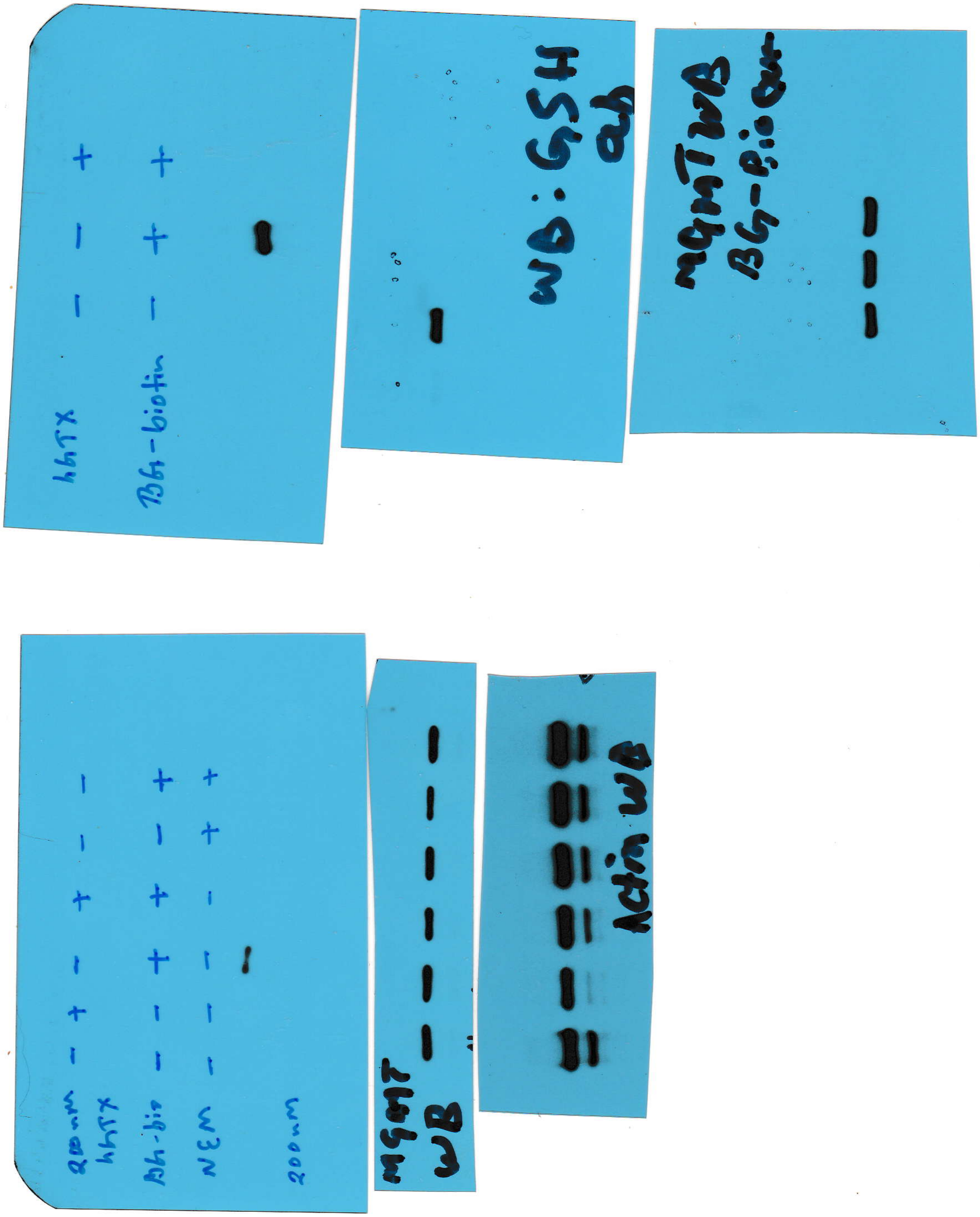

Figure S5. Whole blot of Figure 7 AB

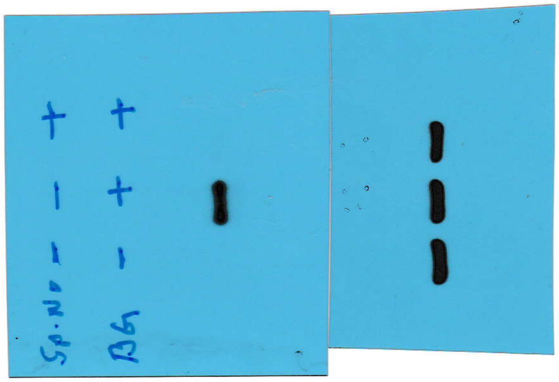

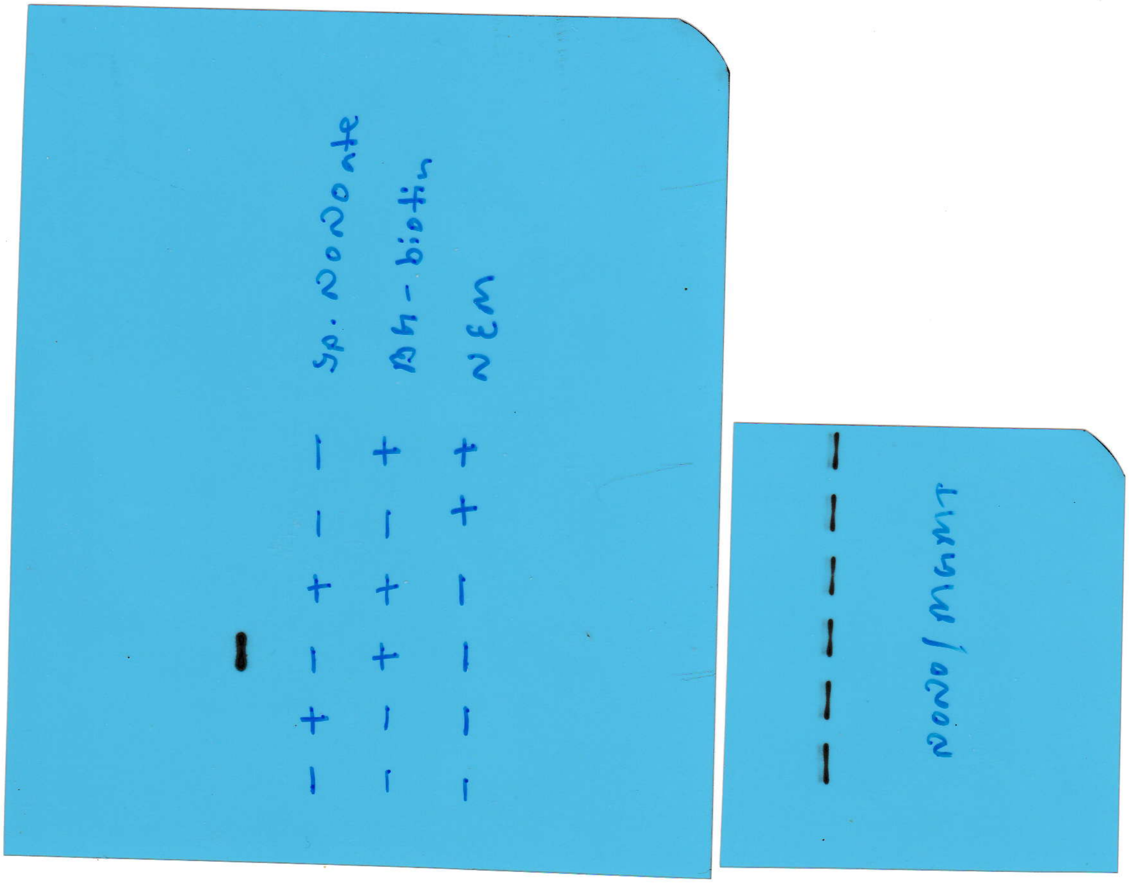

Figure S6. Whole blot of Figure 12C

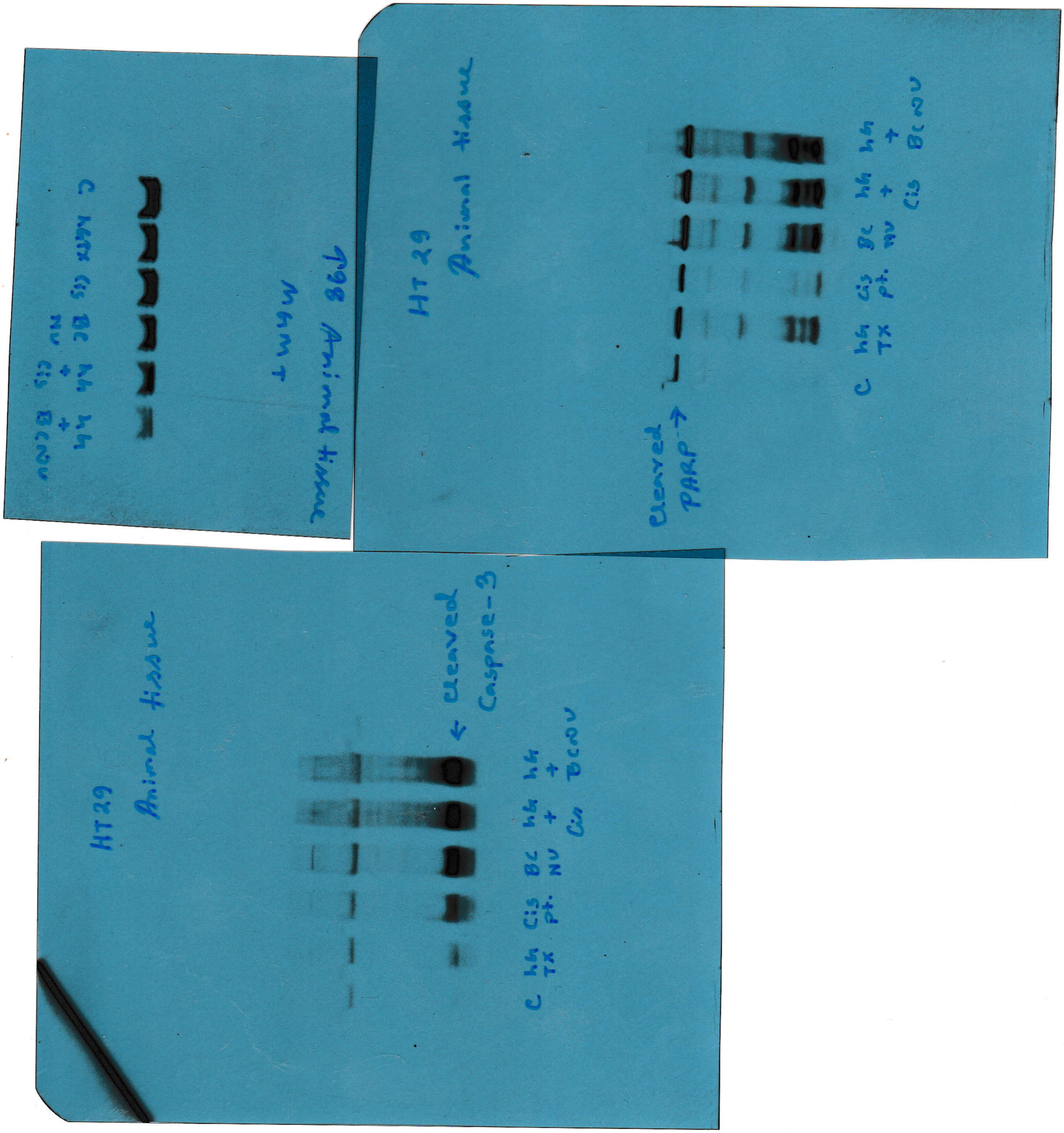

Figure S7. Whole blot of Figure 13C

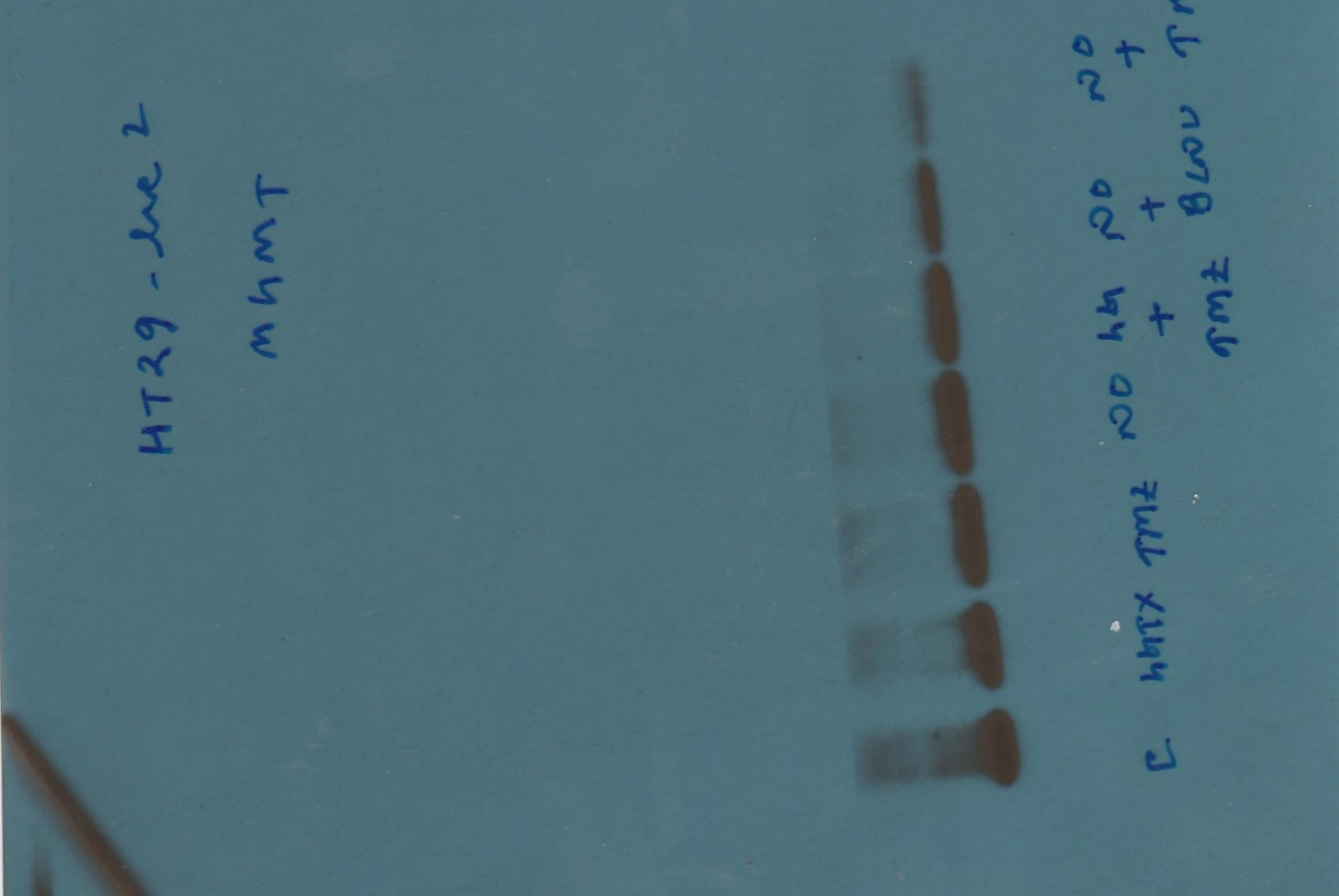

Figure S8. Whole blot of Figure 16D

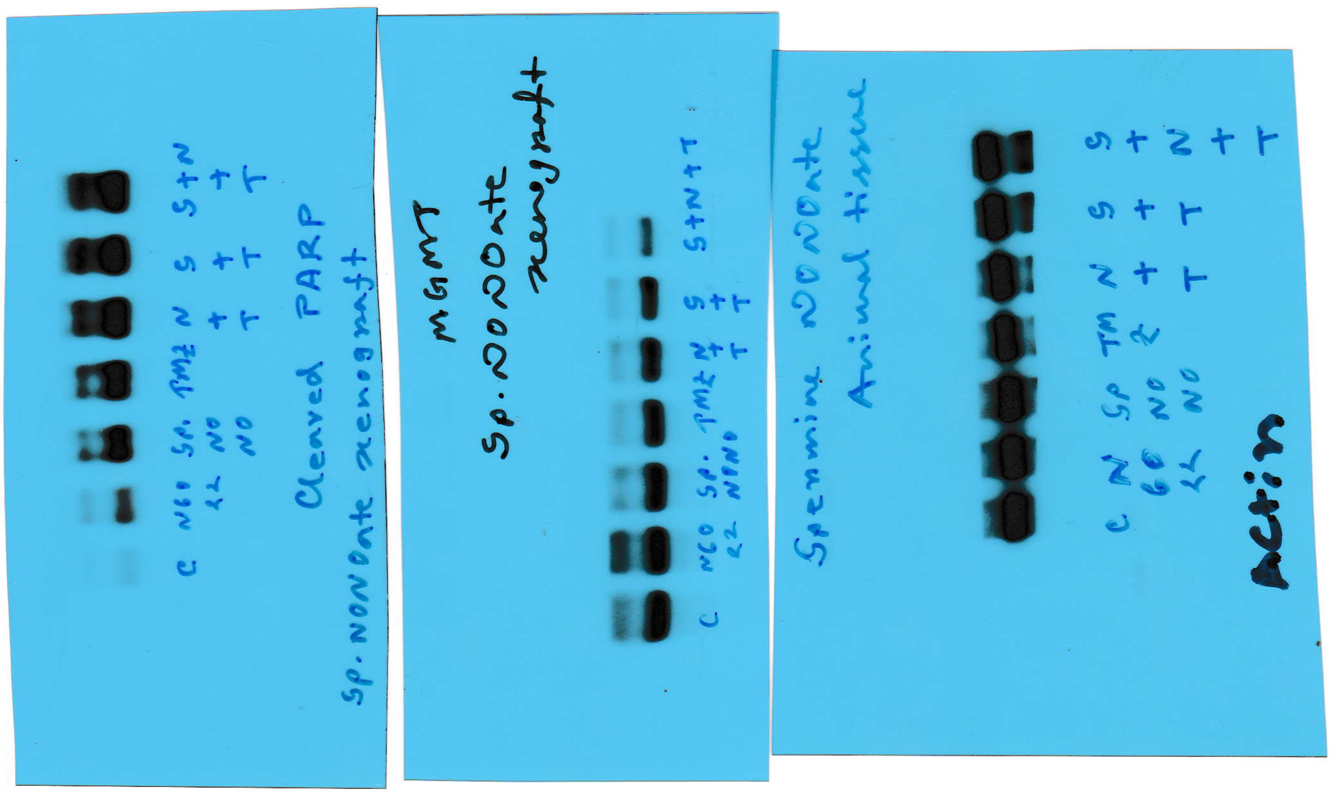

Figure S9. Whole blot of Figure 11 DEFG

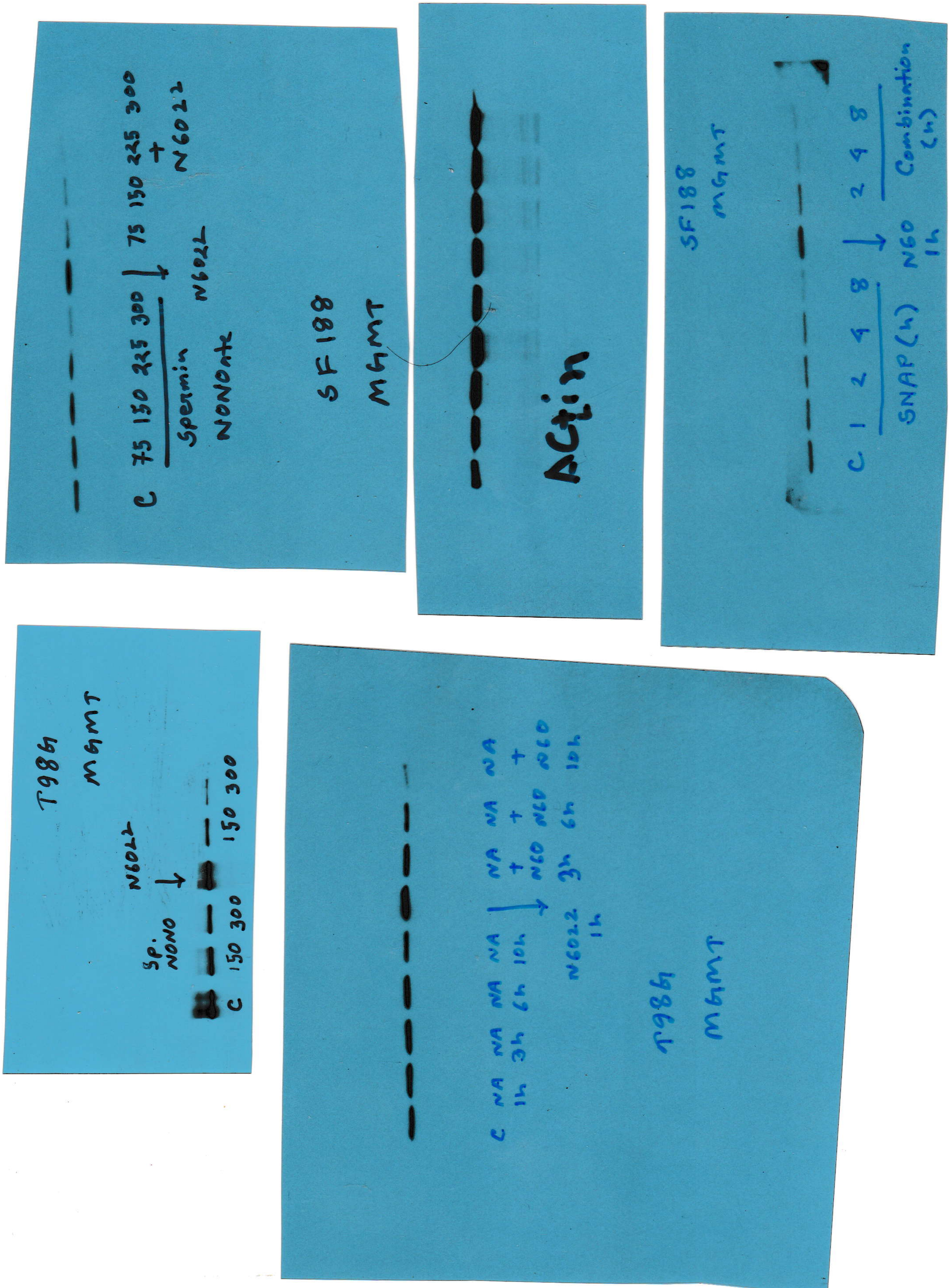

Supplement: Supplementary file 1 [file diseases-13-00032-s001.zip › diseases-3177593-Supplementary.pdf]
